# Supplementary material for: Carneusones A-F, Benzophenone Derivatives from Sponge-Derived Fungus Aspergillus carneus GXIMD00543
Source: Mar Drugs. 2024 Jan 25;22(2):63. doi: 10.3390/md22020063 (PMC10890008; doi:10.3390/md22020063)
Supplement: Supplementary file 1 [file marinedrugs-22-00063-s001.zip › marinedrugs-2826008-supplementary.pdf]

Supporting Information

Carneusones A-F, benzophenone derivatives from sponge-derived fungus *Aspergillus carneus* GXIMD00543

Chun-Ju Lu [†], Li-Fen Liang [†], Geng-Si Zhang, Hai-Yan Li, Chun-Qing Fu, Qin Yu, Dong-Mei Zhou, Zhi-Wei Su, Kai Liu, Cheng-Hai Gao, Xin-Ya Xu*, Yong-Hong Liu*

Institute of Marine Drugs/Guangxi Key Laboratory of Marine Drugs, Guangxi University of Chinese Medicine, Nanning 530200, P. R. China; luchunjv@163.com (C.-J.L.); 15277833592@163.com (L.-F.L.); 15534445495@163.com (G.-S.Z.); lihaiyan12368@163.com (H.-Y.L.); 18378464907@163.com (C.-Q.F.); 19862354152@163.com (Q.Y.); zhoudm@gxtcmu.edu.cn (D.-M.Z.); suzw1454@126.com (Z.-W.S.); liuk@gxtcmu.edu.cn (K.L.); gaoch@gxtcmu.edu.cn (C.-H.G.)

* xyxu@gxtcmu.edu.cn (X.-Y.X.); yonghongliu@scsio.ac.cn (Y.-H.L.)

† These authors contributed equally to this work.

Table of Contents

Figure S1. The sponge *Haliclona* sp., colonies and the ITS rRNA sequences data of sponge-derived fungus *Aspergillus carneus* GXIMD00543 3

Figure S2. ^{1}H NMR spectrum of **1** 3

Figure S3. ^{13}C NMR spectrum of **1** 4

Figure S4. HSQC spectrum of **1**.. 4

Figure S5. HMBC spectrum of **1**.. 5

Figure S6. HRESIMS spectrum of **1** 5

Figure S7. ^{1}H NMR spectrum of **2** 6

Figure S8. ^{13}C NMR spectrum of **2** 6

Figure S9. HSQC spectrum of **2**.. 7

Figure S10. HMBC spectrum of **2**.. 7

Figure S11. HRESIMS spectrum of **2** 8

Figure S12. ^{1}H NMR spectrum of **3** 8

Figure S13. ^{13}C NMR spectrum of **3** 9

Figure S14. HSQC spectrum of **3**.. 9

Figure S15. HMBC spectrum of **3**.. 10

Figure S16. HRESIMS spectrum of **3** 10

Figure S17. ^{1}H NMR spectrum of **4** 11

Figure S18. ^{13}C NMR spectrum of **4** 11

Figure S19. HSQC spectrum of **4**.. 12

Figure S20. HMBC NMR spectrum of **4**.. 12

Figure S21. HRESIMS spectrum of **4** 13

Figure S22. ^{1}H NMR spectrum of **5** 13

Figure S23. ^{13}C NMR spectrum of **5** 14

Figure S24. HSQC spectrum of **5**.. 14

Figure S25. HMBC spectrum of **5**.. 15

Figure S26. NOESY spectrum of **5** 15

Figure S27. HRESIMS spectrum of **5** 16

Figure S28. ^{1}H NMR spectrum of **6** 16

Figure S29. ^{13}C NMR spectrum of **6** 17

Figure S30. DEPT135 spectrum of **6** 17

Figure S31. HSQC spectrum of **6** ... 18

Figure S32. HMBC spectrum of **6** ... 18

Figure S33. NOESY spectrum of **6** ... 19

Figure S34. HRESIMS spectrum of **6** ... 19

Figure S35. The optimized structures of conformers **5a-5d** in MeOH at B3LYP/6-31G(d,p) level ... 20

Table S1. The Cartesian coordinates of optimized structure of (10*S*,11*S*,12*S*)-**5a** ... 20

Table S2. The Cartesian coordinates of optimized structure of (10*R*,11*R*,12*R*)-**5b** ... 21

Table S3. The Cartesian coordinates of optimized structure of (10*S*,11*R*,12*S*)-**5c** ... 23

Table S4. The Cartesian coordinates of optimized structure of (10*R*,11*S*,12*R*)-**5d** ... 24

Figure S36. DP4+ probabilities (%) for configurations (10*S*,11*S*,12*S*)-**5a** (isomer 1), (10*R*,11*R*,12*R*)-**5b** (isomer 2), (10*S*,11*R*,12*S*)-**5c** (isomer 3), and (10*R*,11*S*,12*R*)-**5d** (isomer 4) ... 25

Figure S37. The optimized structure of conformer (10*S*, 12*S*)-**6a** in MeOH at B3LYP/6-31G(d,p) level ... 26

Table S5. The Cartesian coordinates of optimized structure of (10*S*, 12*S*)-**6a** ... 26

Spectroscopic data and chemical structures of compounds **7–13** ... 27

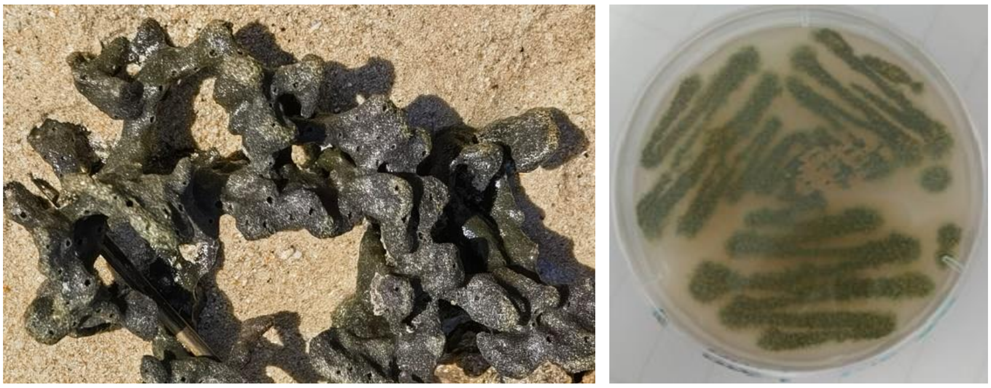

TACCTGATCCGAGGTCACCTGAAGAAAAATGGTTGGACGTCGGCTGGCGCCCGGCCGGCCCTAAATCGAGC
GGGTGACAAAGCCCCATACGCTCGAGGACCGGACACGGTGCCGCCGCTGCCTTTCGGGCCCGTCCCCGG
GGGGGACGACGACCCAACACACAAGCCGGGCTTGATGGGCAGCAATGACGCTCGGACAGGCATGCCCCC
CGGAATGCCAGGGGGCGCAATGTGCGTTCAAAGACTCGATGATTCACTGAATTCTGCAATTCACATTACTTAT
CGCAGTTCGCTGCGTTCTTCATCGATGCCGGAACCAAGAGATCCATTGTTGAAAGTTTTGACTGATTTTATAT
TCAGACTCAGACTGCATCACTCTCAGGCATGAAGTTCAGTAGTCCCCGGCGGCTCGCCCCCGAGAGGACTC
CCCGCCGAAGCAACAGTGTTAGGTAGTCACGGGTGGGAGGTTGGGCGCCCGGAGGCAGCCCGCACTCAG
TAATGATCCTTCCGCAGGTTCACCCTACGGAAG

Figure S1. The sponge *Haliclona* sp. (left), colonies (right) and the ITS rRNA sequences data of sponge-derived fungus *Aspergillus carneus* GXIMD00543

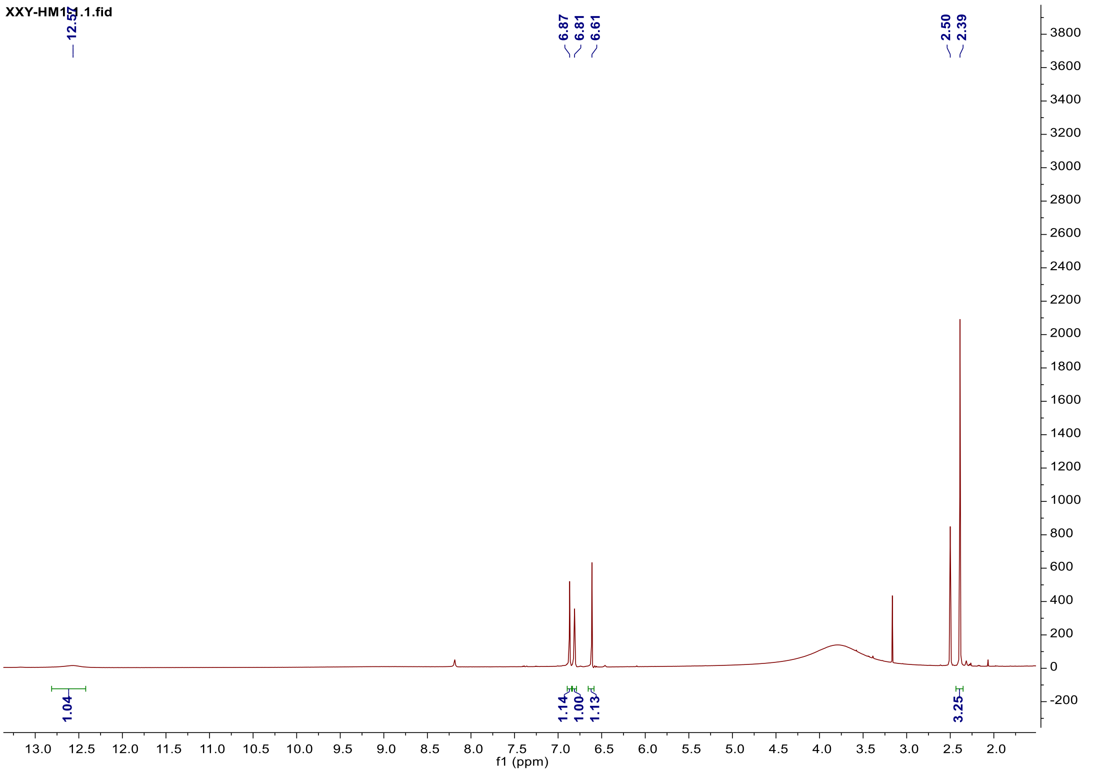

Figure S2. ^{1}H NMR spectrum of **1**

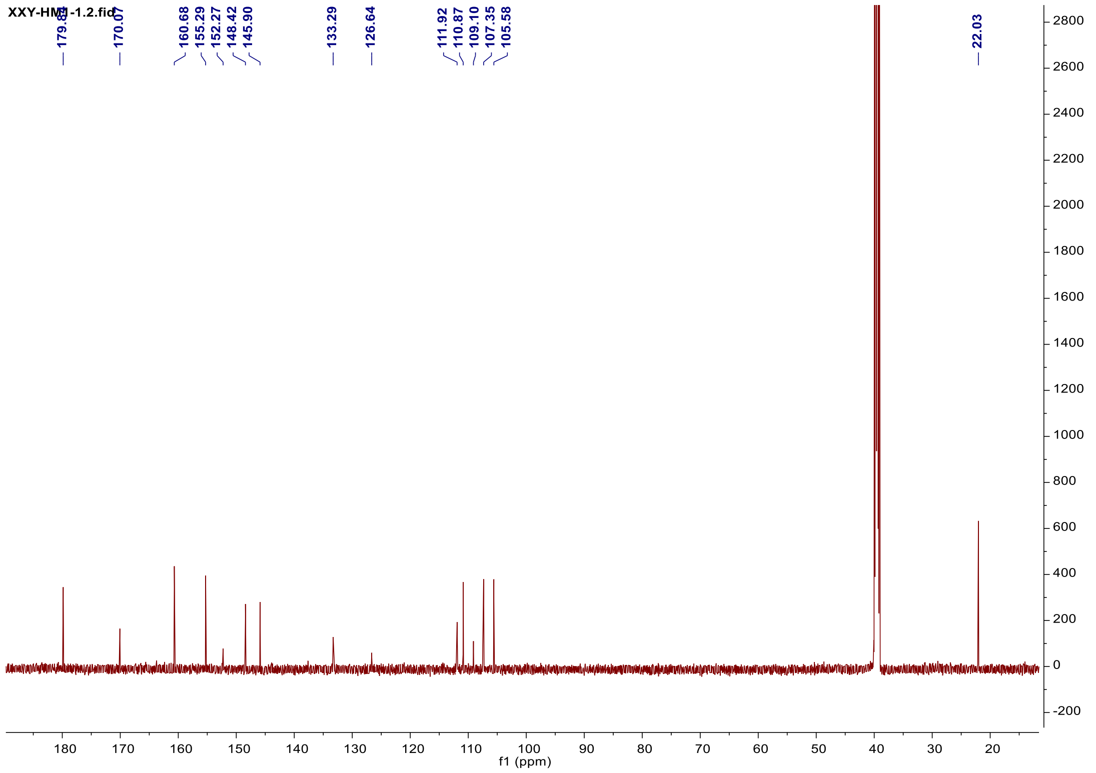

Figure S3. ^{13}C NMR spectrum of **1**

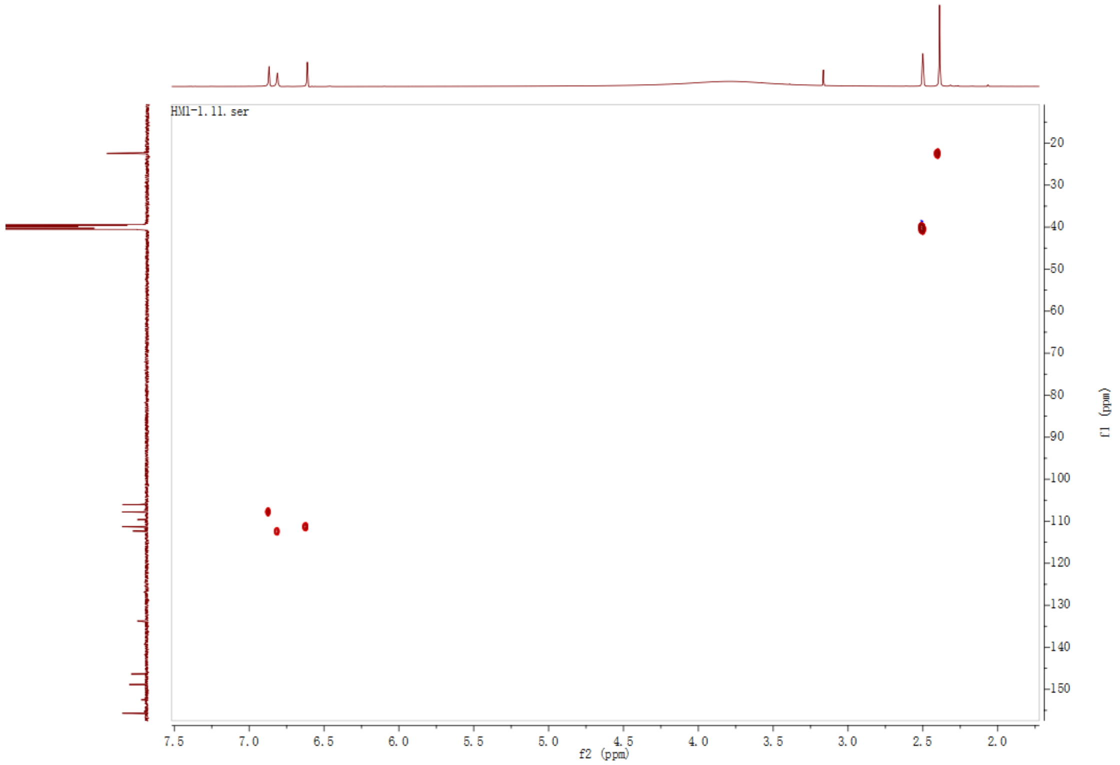

Figure S4. HSQC spectrum of **1**

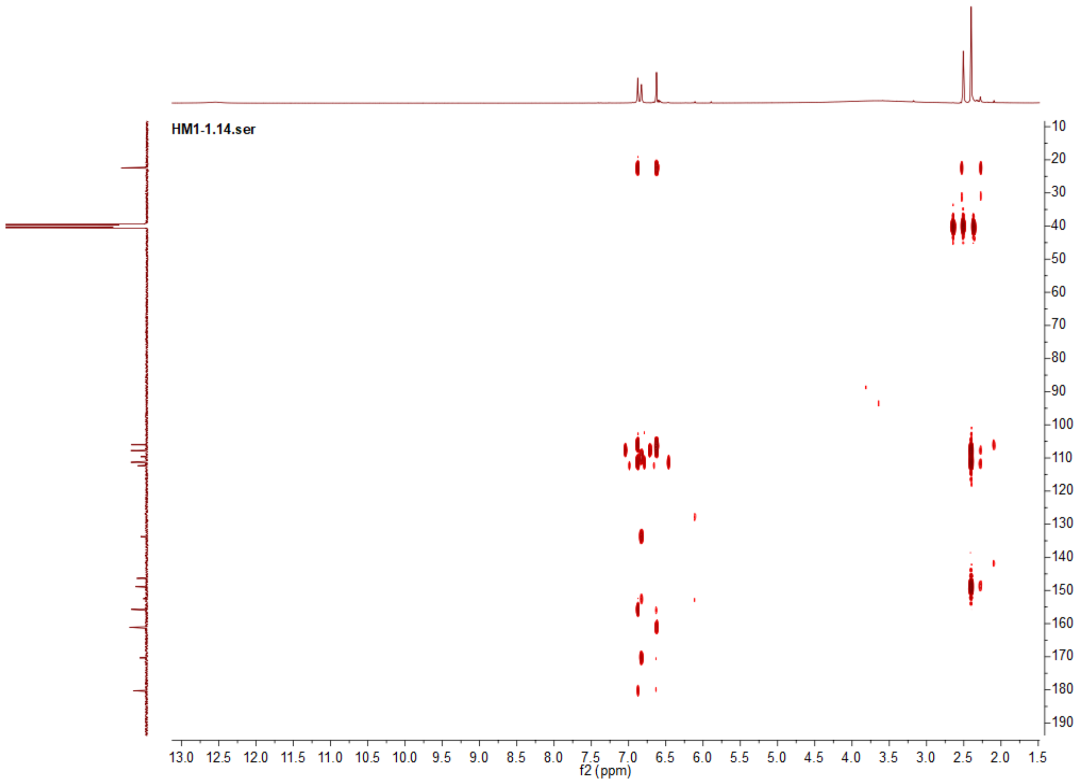

Figure S5. HMBC spectrum of **1**

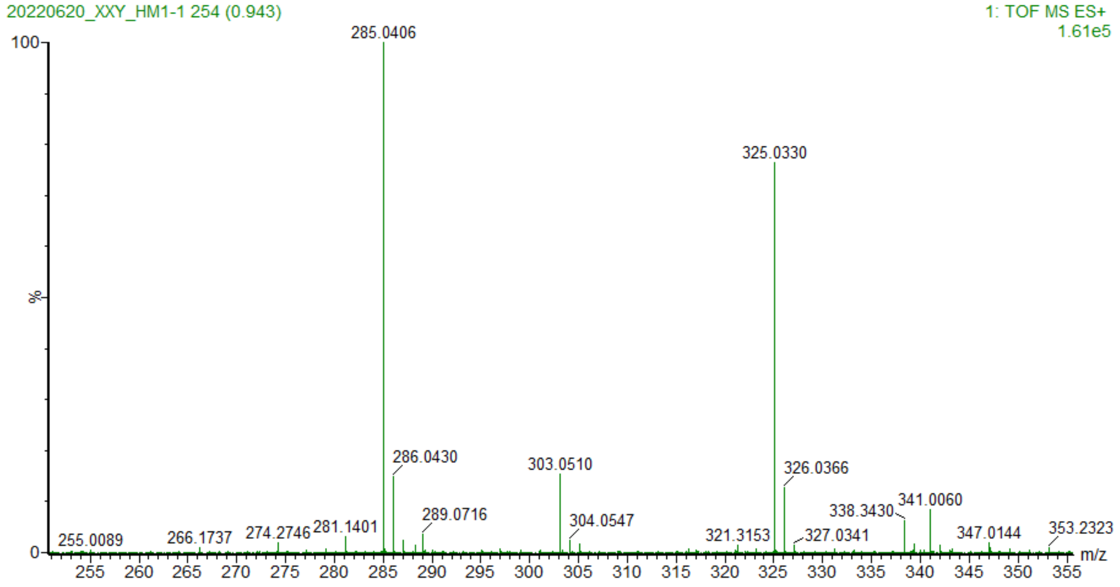

Figure S6. HRESIMS spectrum of **1**

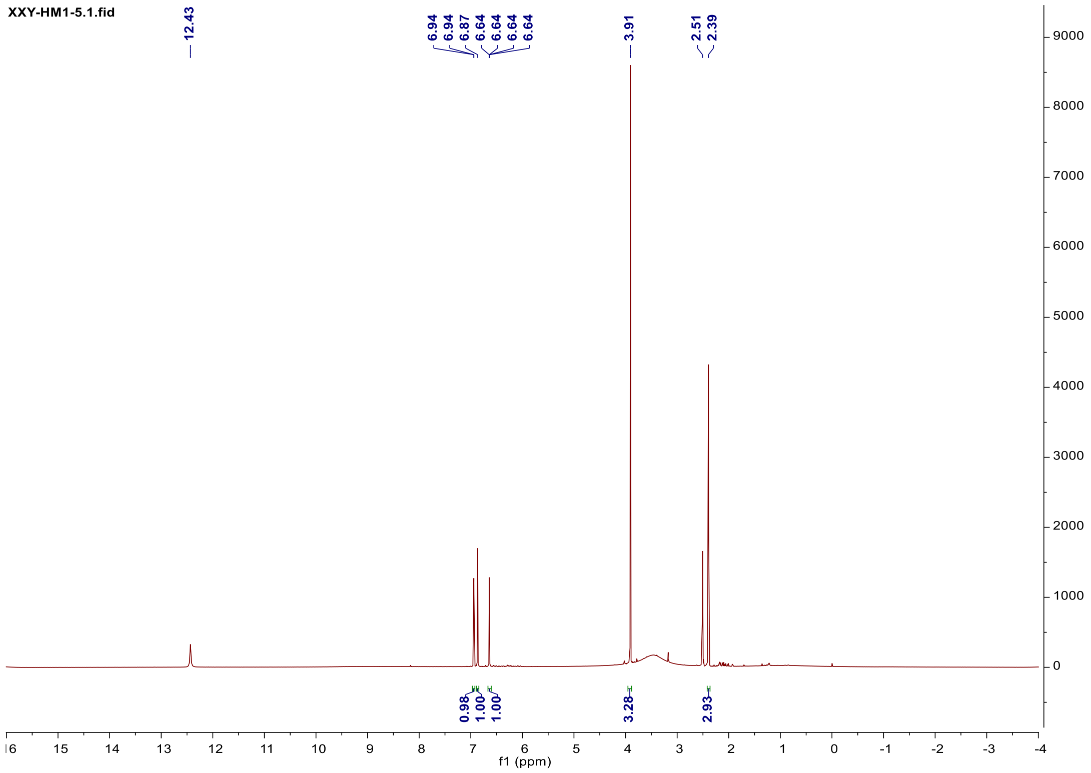

Figure S7. ^{1}H NMR spectrum of **2**

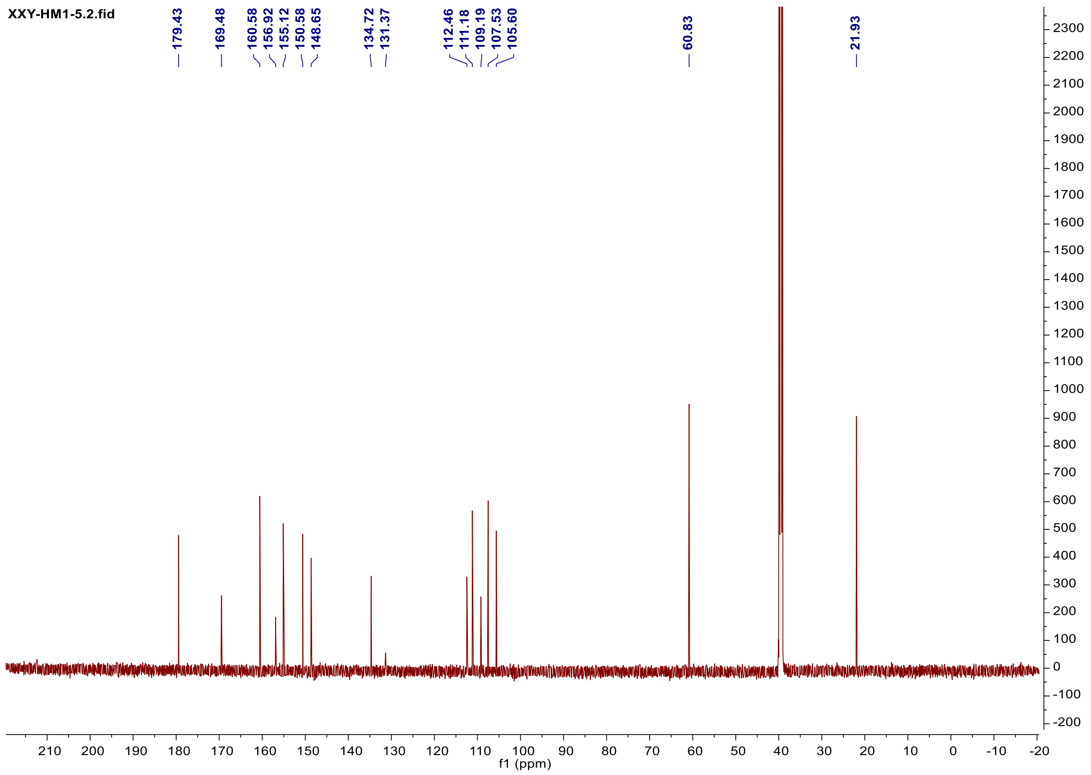

Figure S8. ^{13}C NMR spectrum of **2**

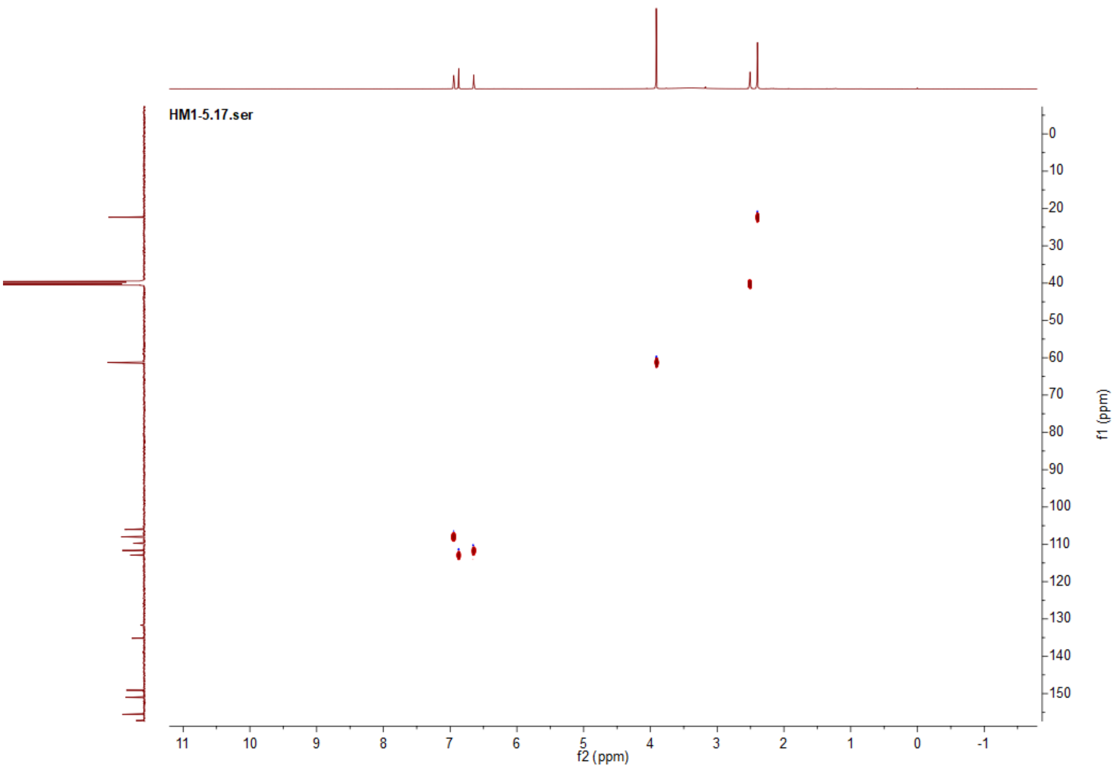

Figure S9. HSQC spectrum of **2**

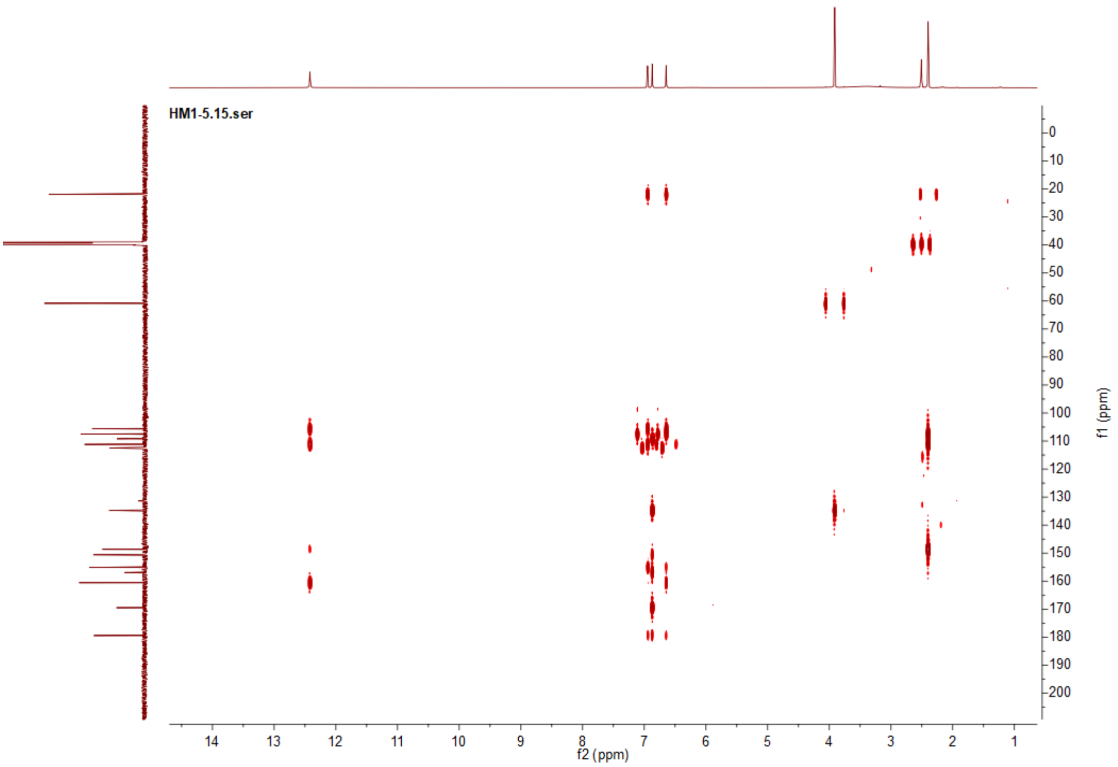

Figure S10. HMBC spectrum of **2**

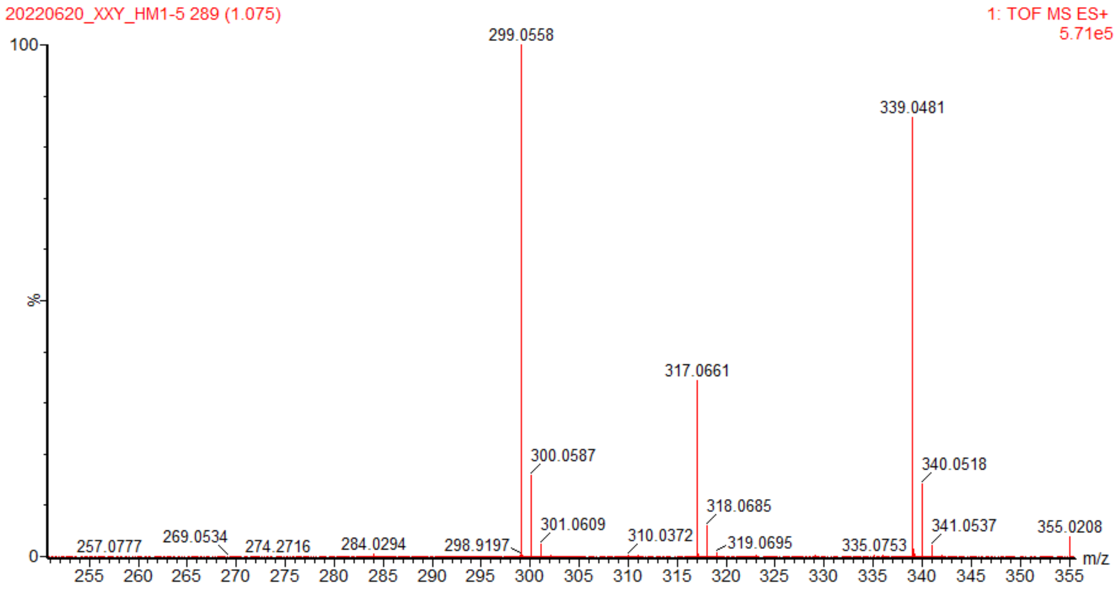

Figure S11. HRESIMS spectrum of **2**

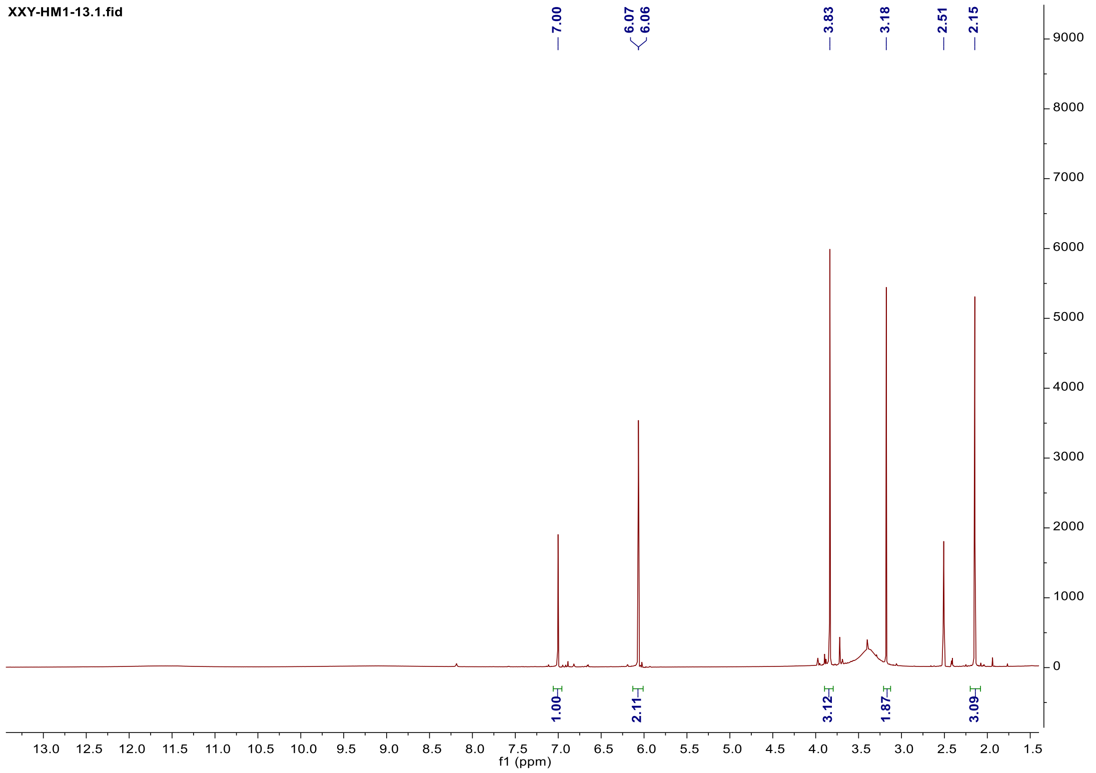

Figure S12. ^{1}H NMR spectrum of **3**

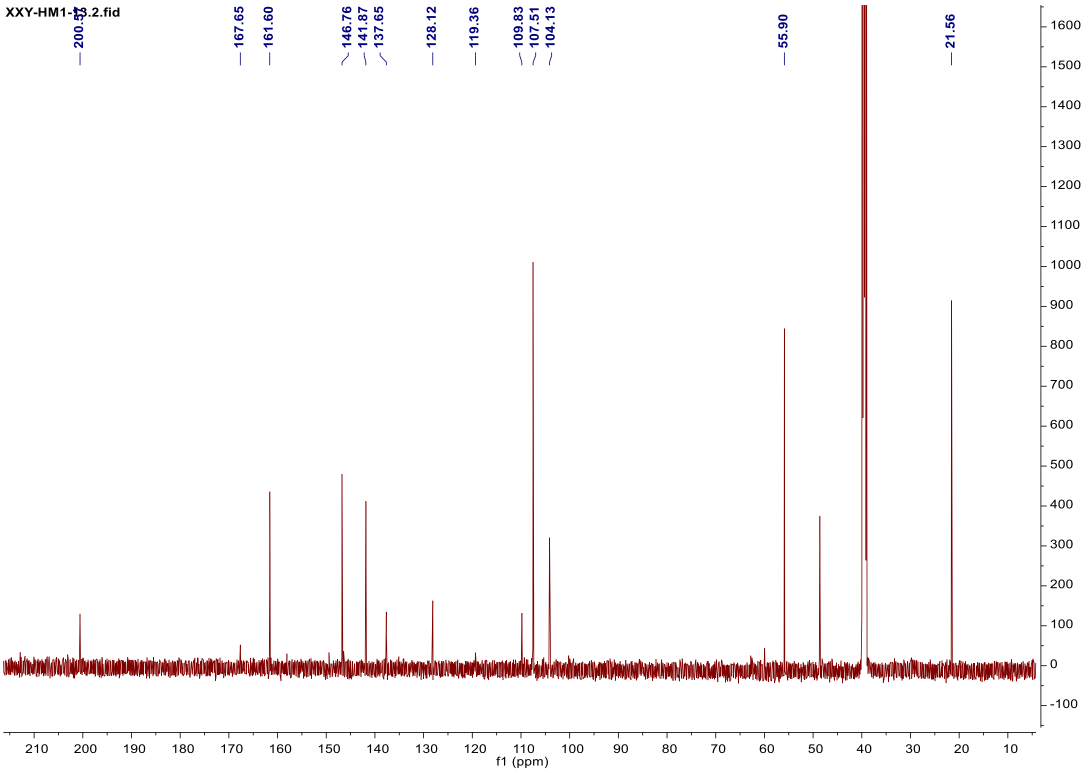

Figure S13. ^{13}C NMR spectrum of **3**

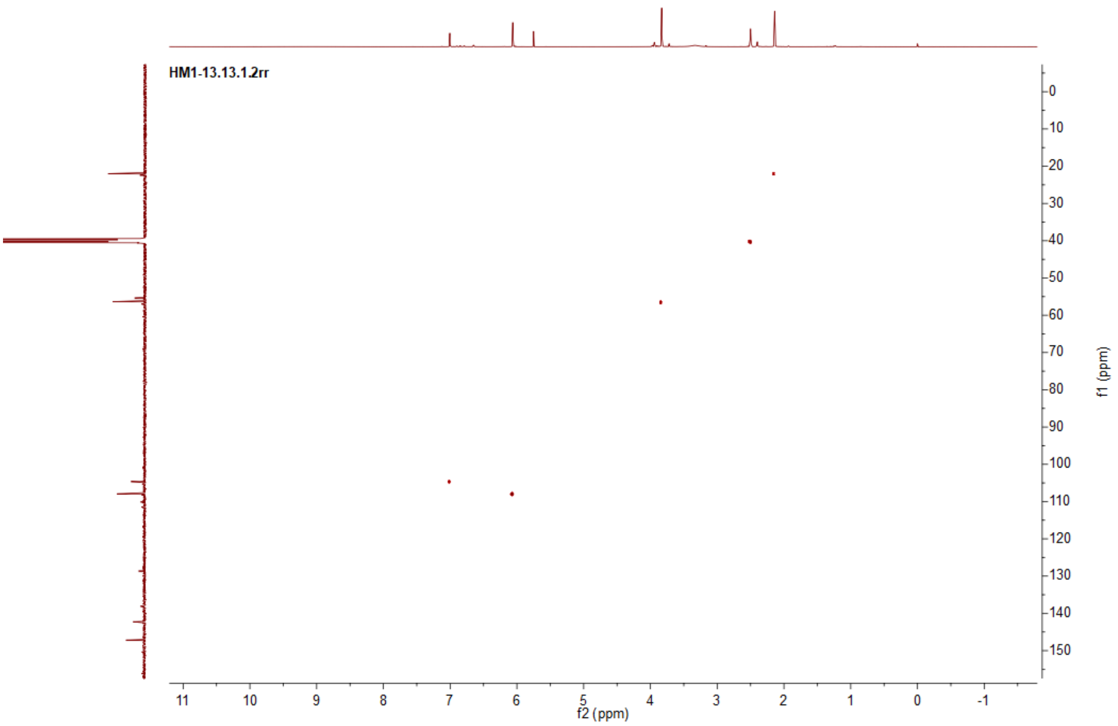

Figure S14. HSQC spectrum of **3**

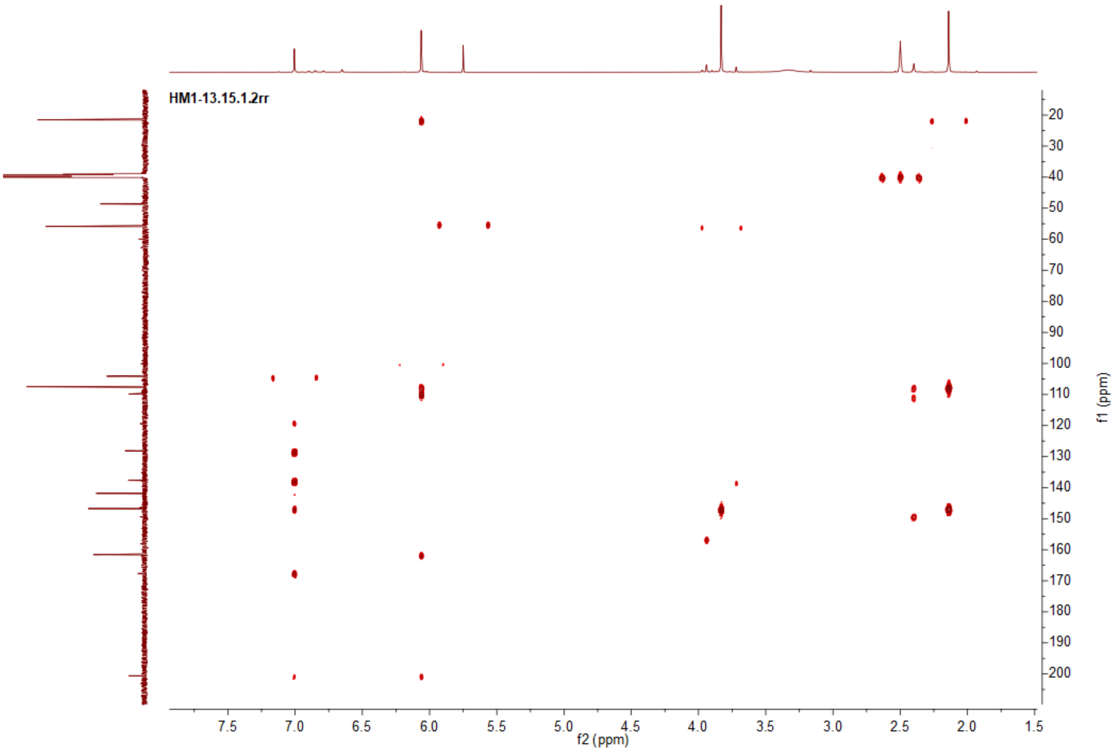

Figure S15. HMBC spectrum of **3**

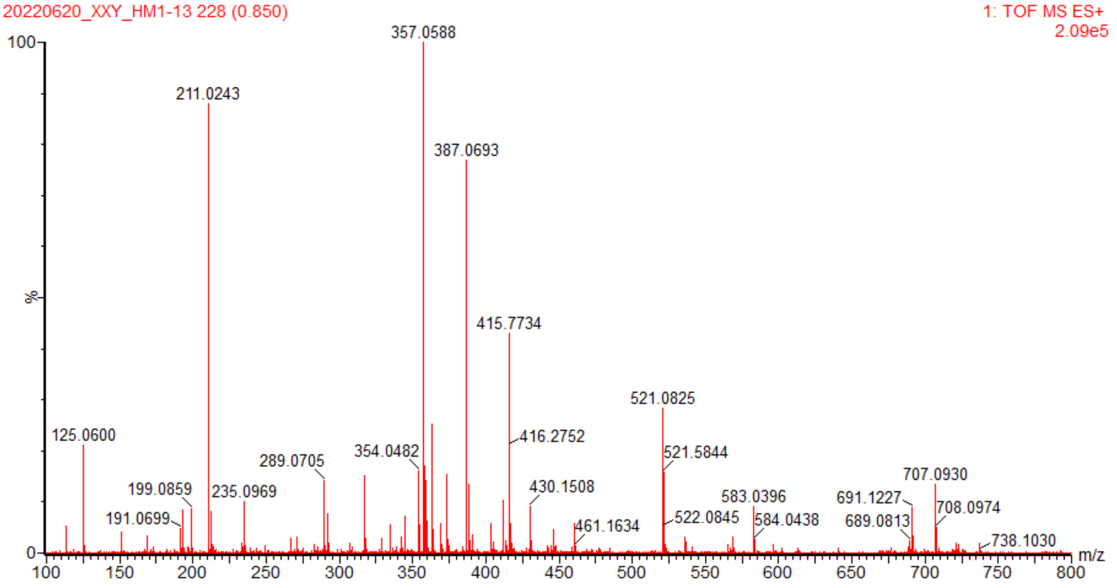

Figure S16. HRESIMS spectrum of **3**

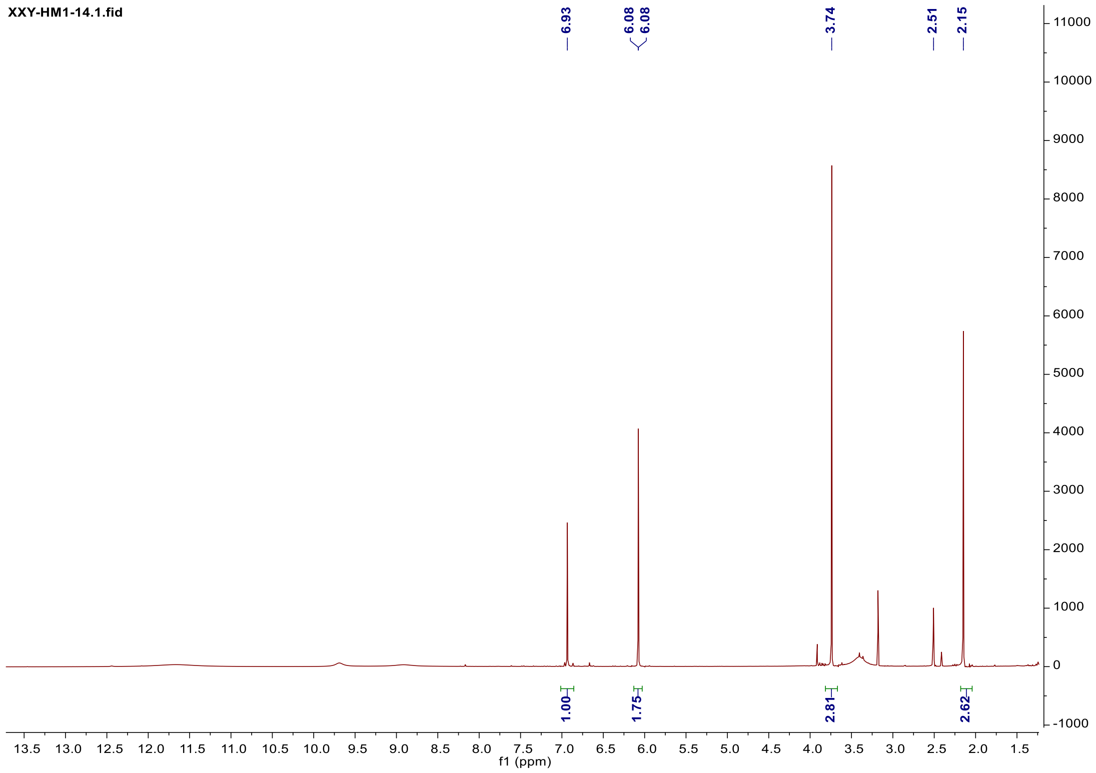

Figure S17. ^{1}H NMR spectrum of **4**

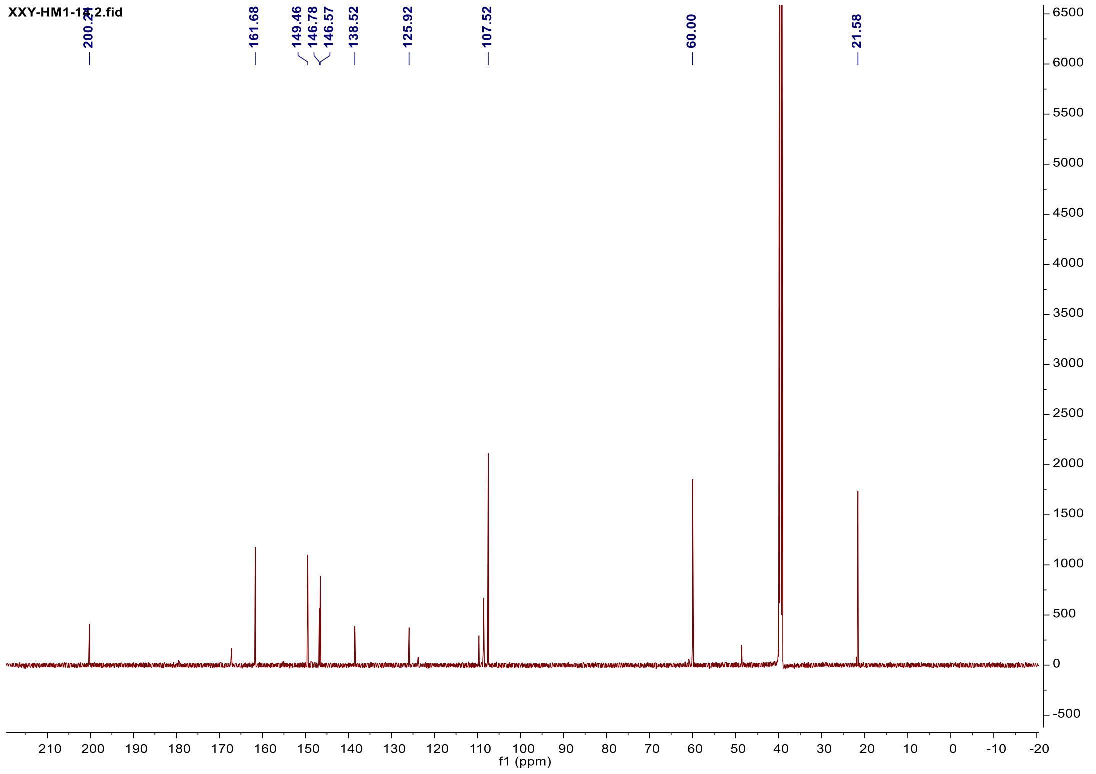

Figure S18. ^{13}C NMR spectrum of **4**

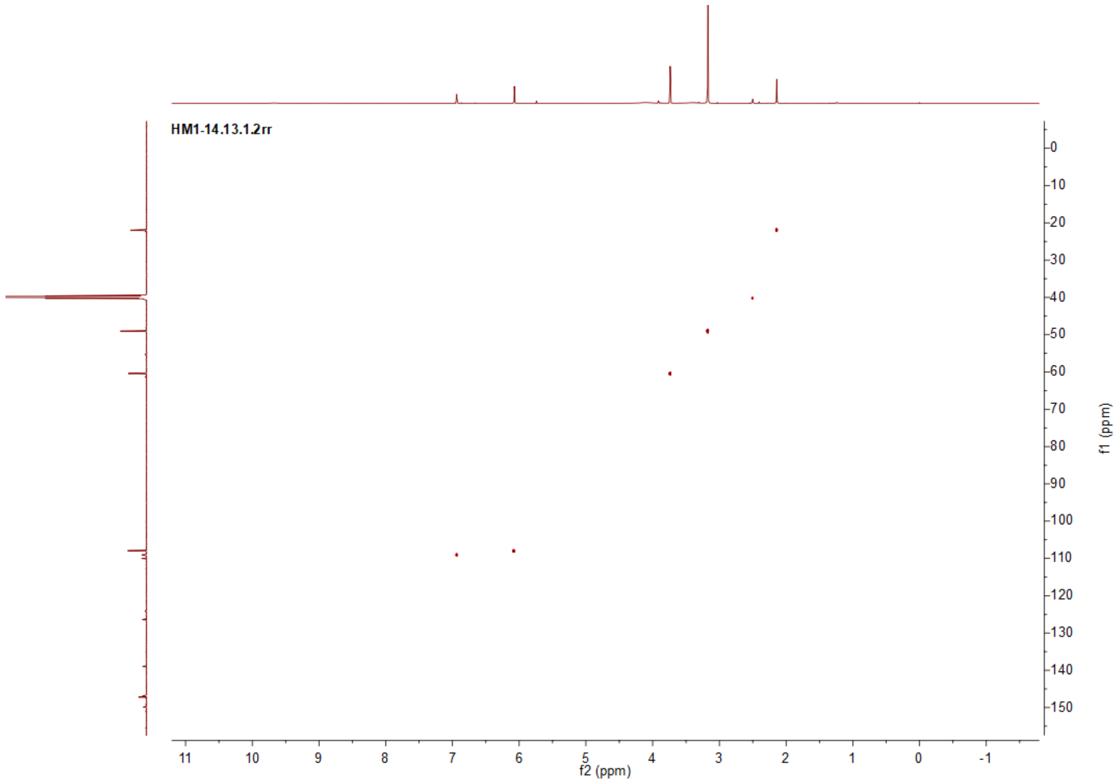

Figure S19. HSQC spectrum of **4**

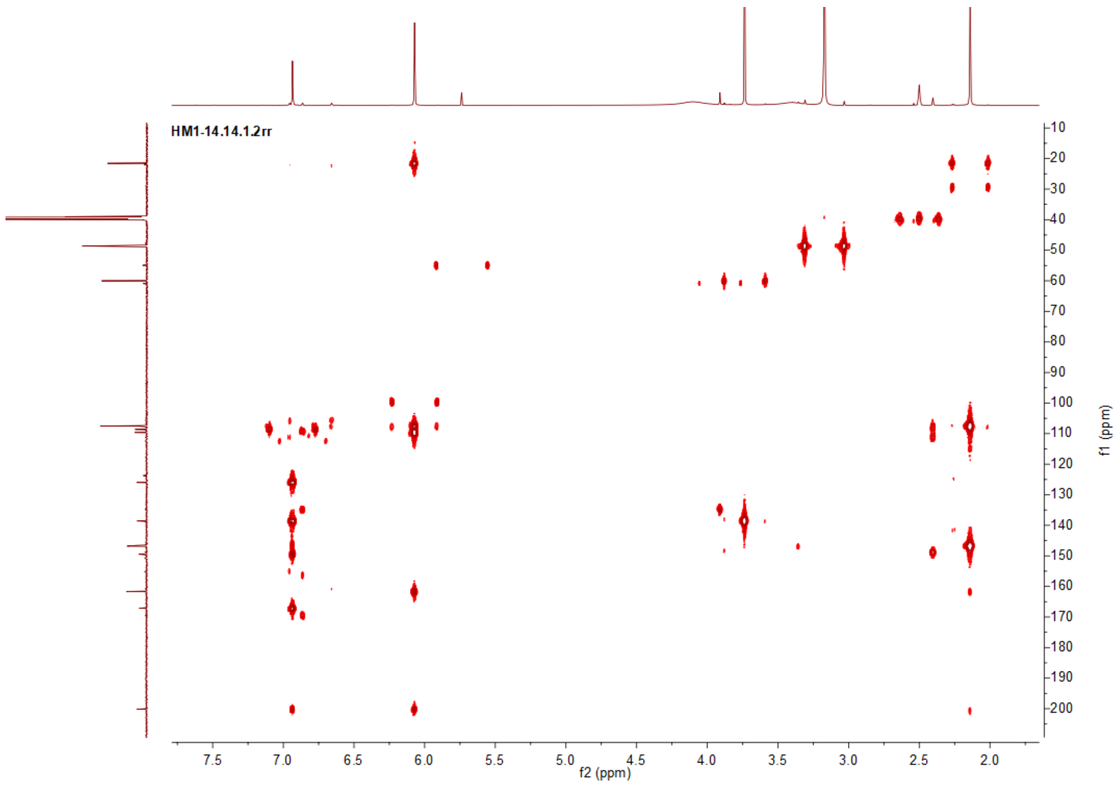

Figure S20. HMBC NMR spectrum of **4**

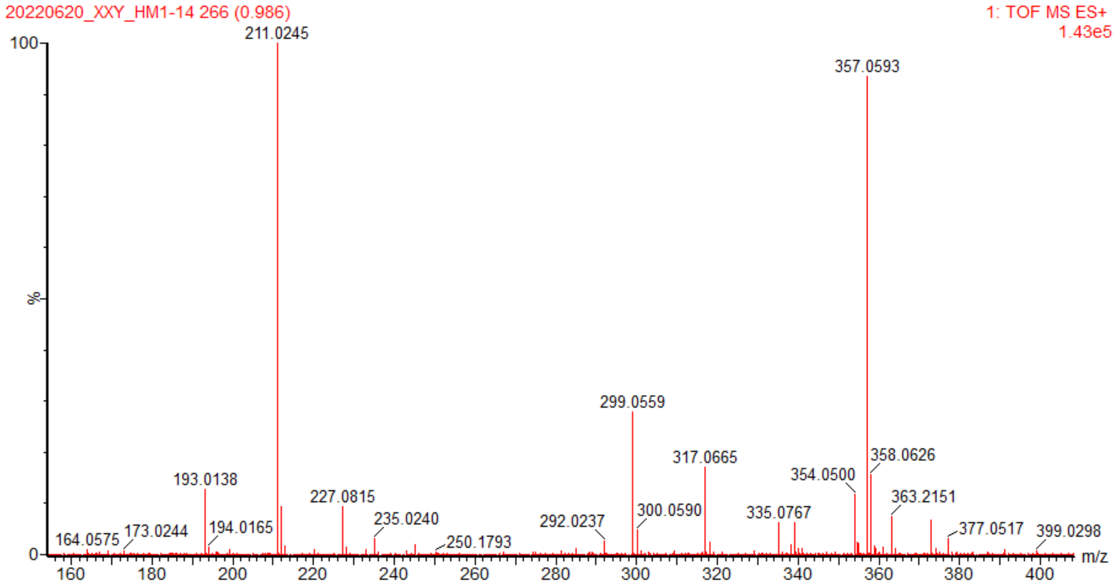

Figure S21. HRESIMS spectrum of **4**

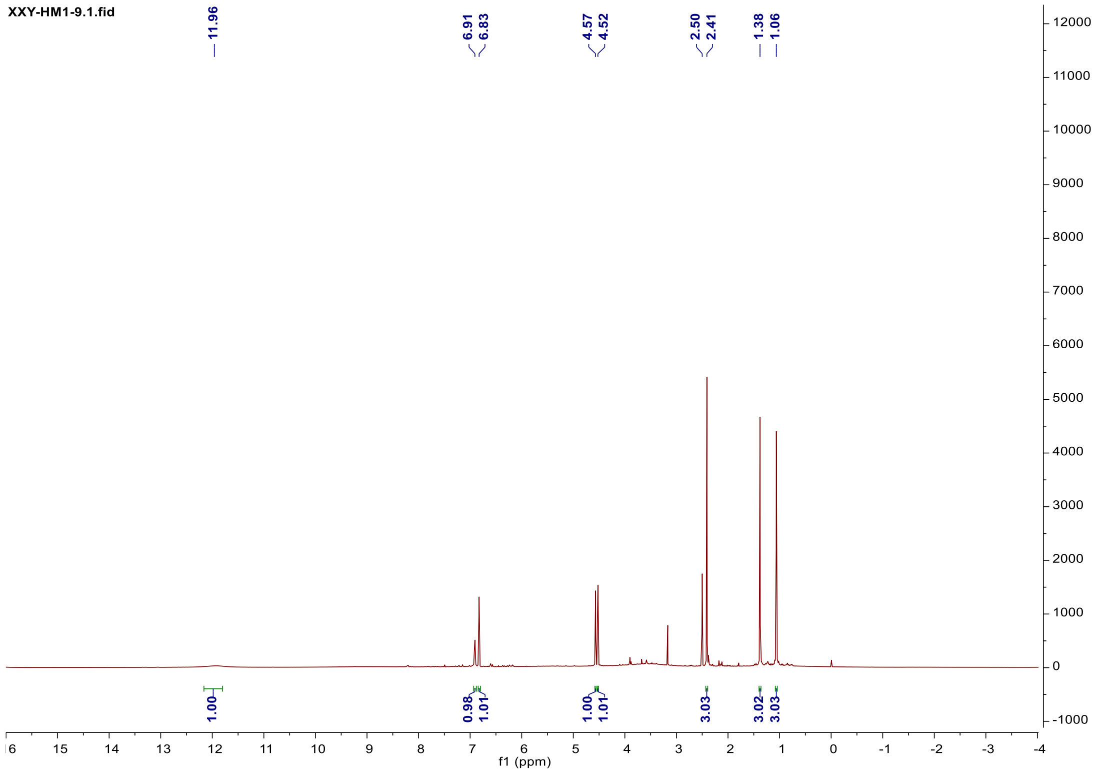

Figure S22. ^{1}H NMR spectrum of **5**

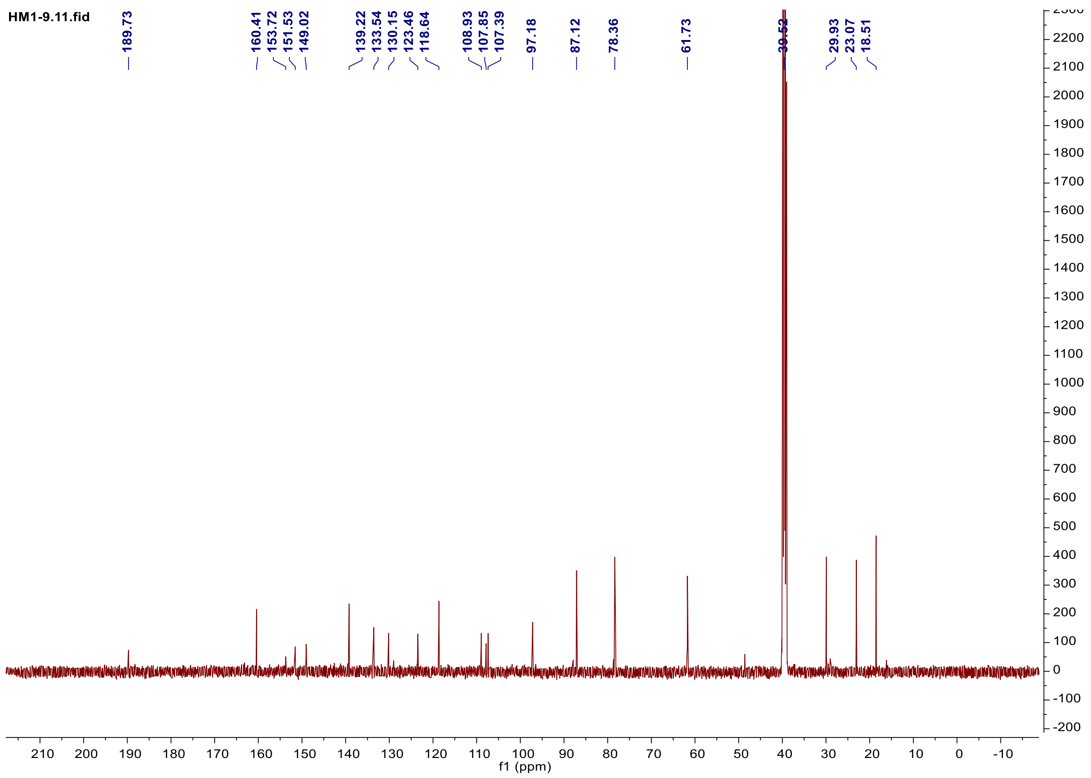

Figure S23. ^{13}C NMR spectrum of **5**

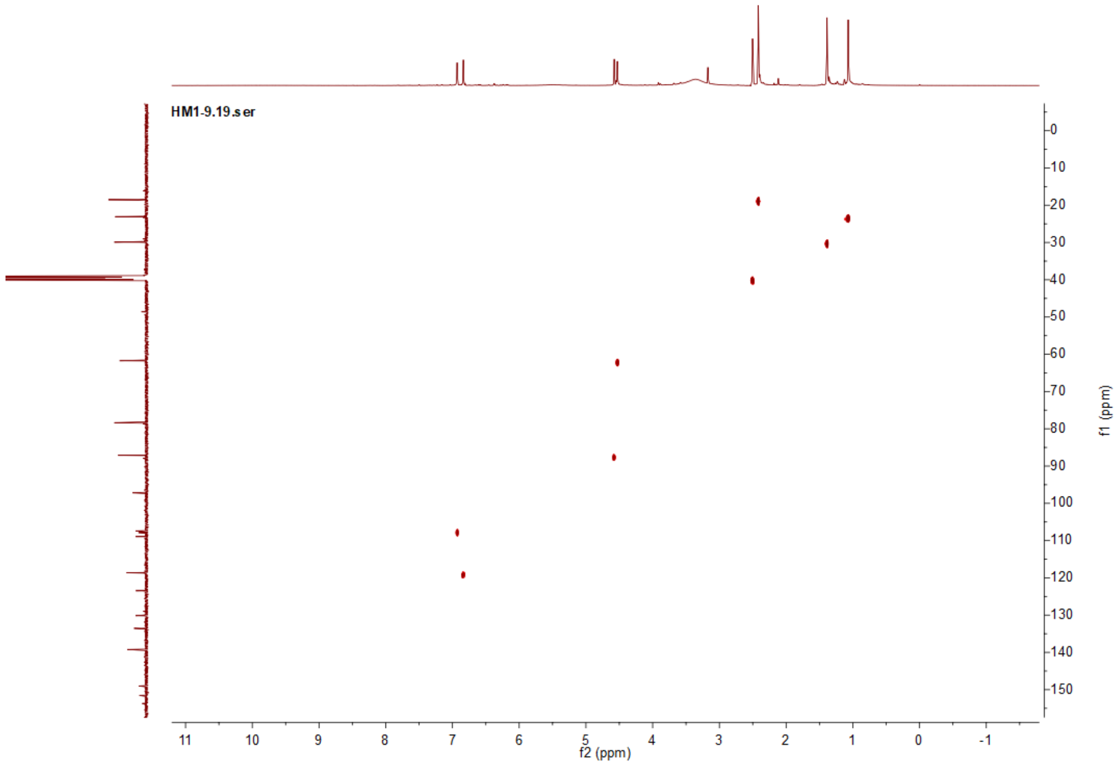

Figure S24. HSQC spectrum of **5**

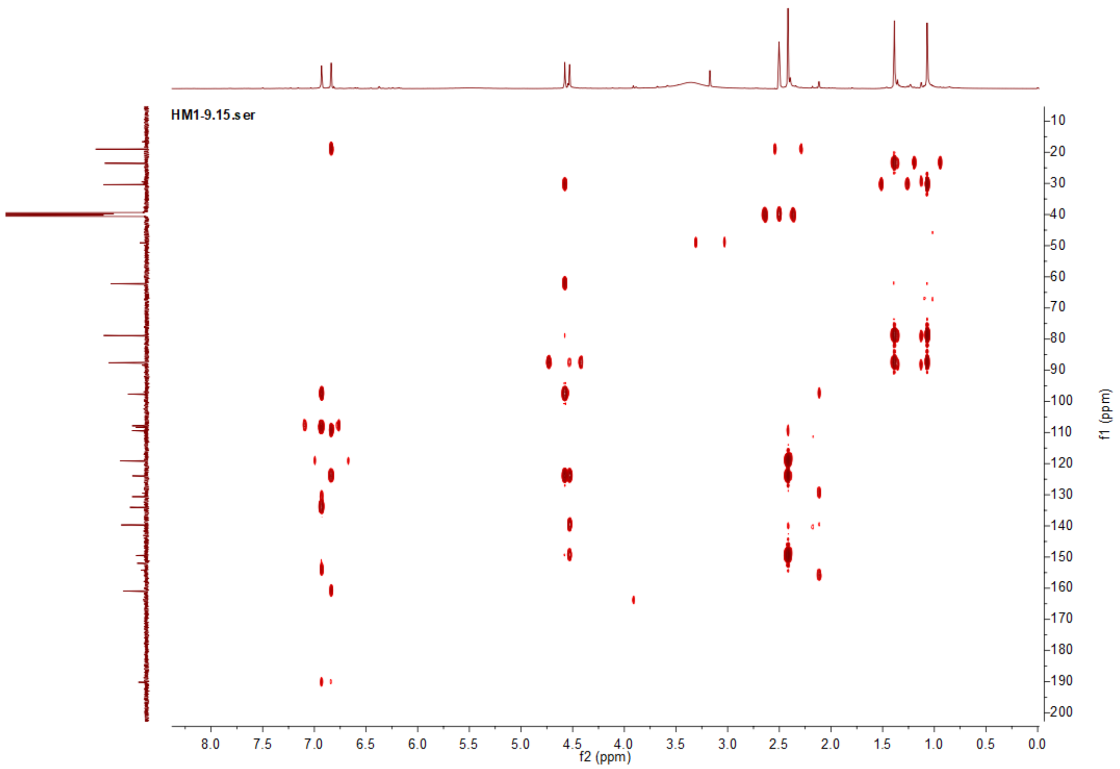

Figure S25. HMBC spectrum of **5**

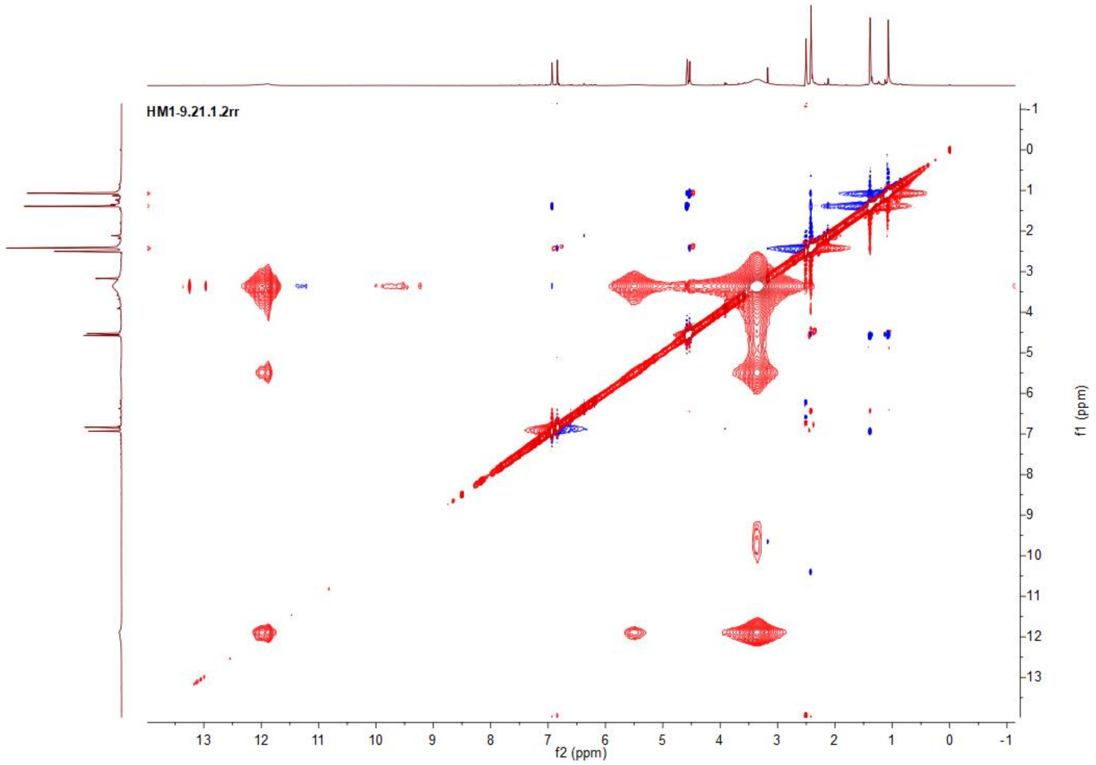

Figure S26. NOESY spectrum of **5**

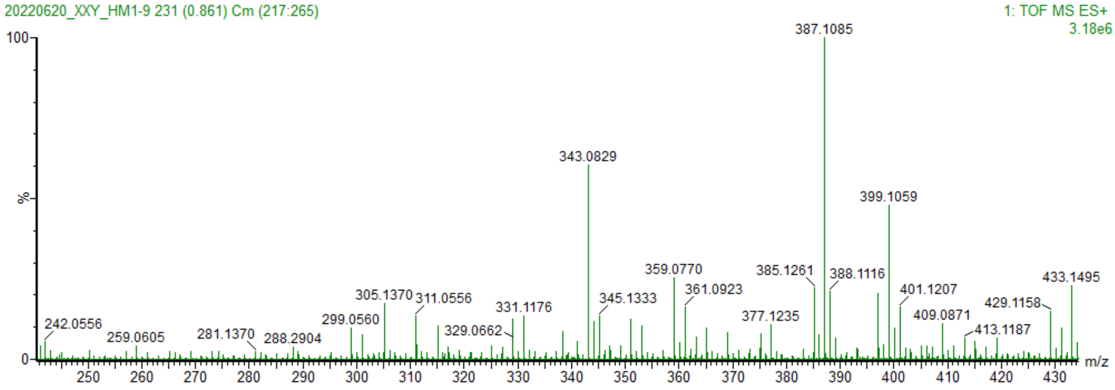

Figure S27. HRESIMS spectrum of **5**

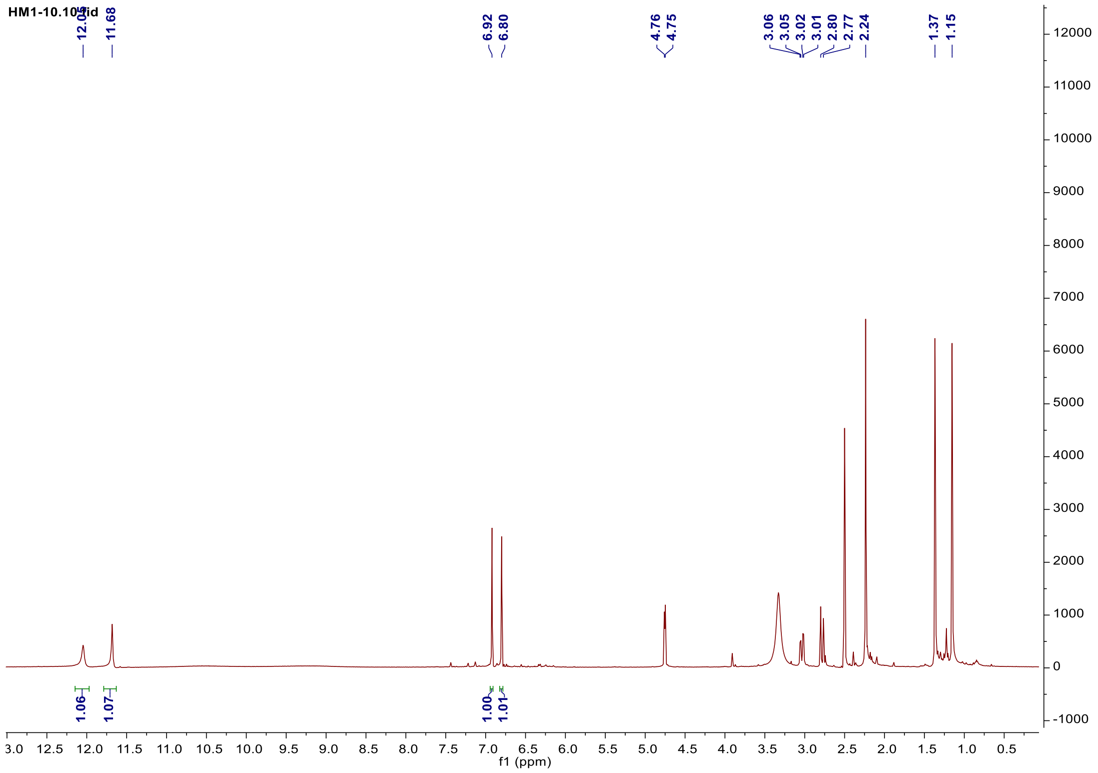

Figure S28. ^{1}H NMR spectrum of **6**

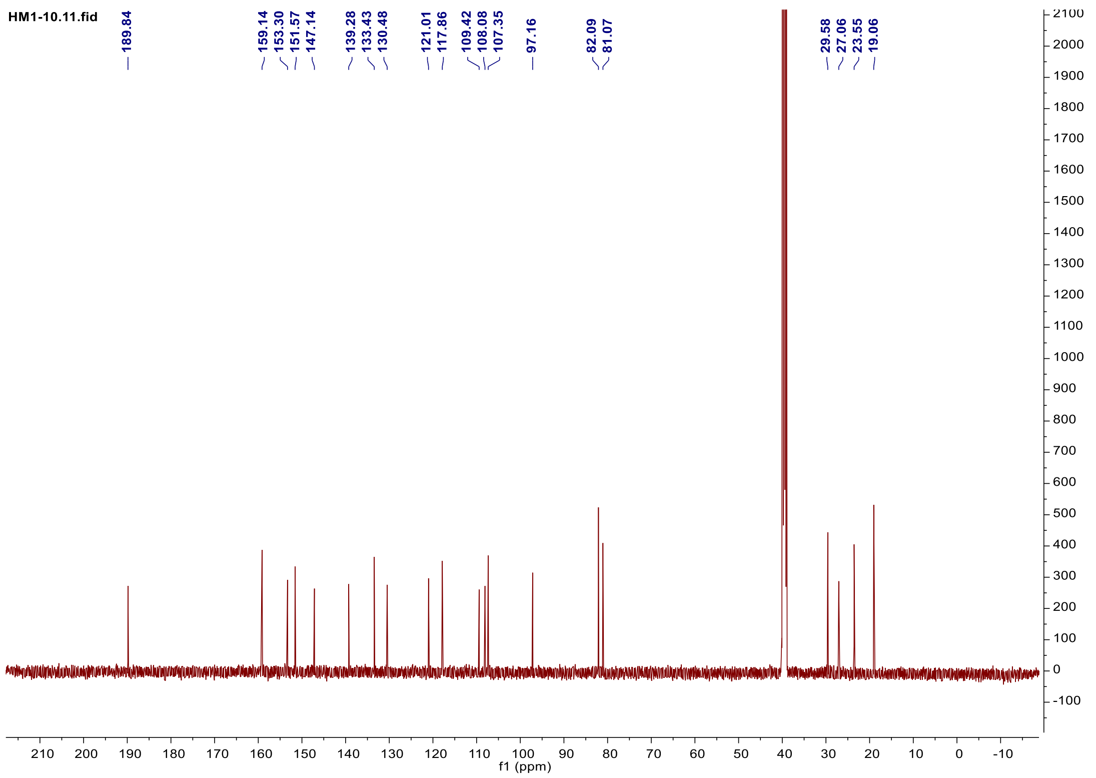

Figure S29. ^{13}C NMR spectrum of **6**

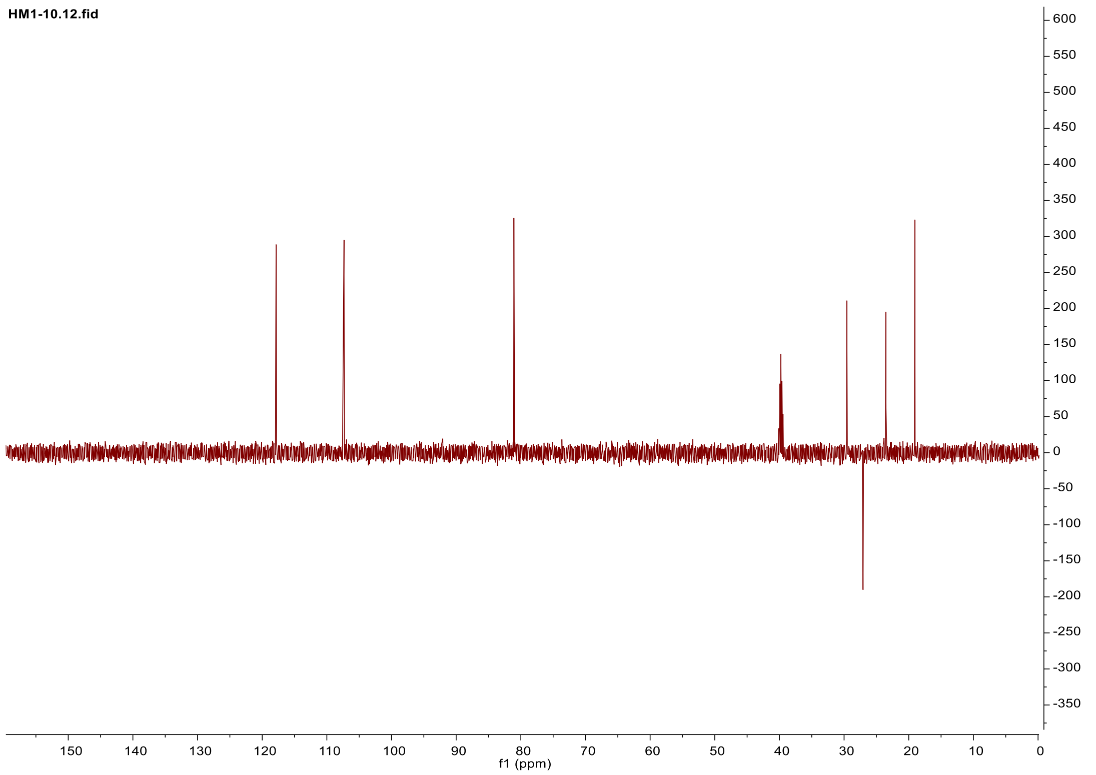

Figure S30. DEPT135 spectrum of **6**

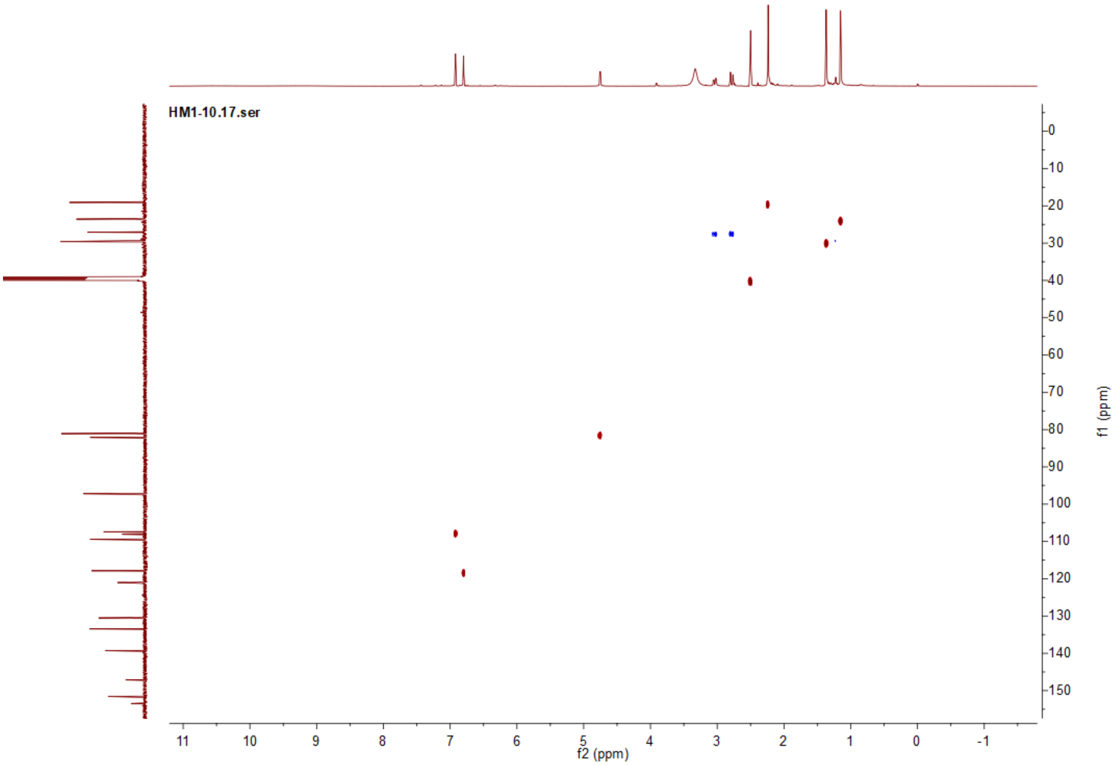

Figure S31. HSQC spectrum of **6**

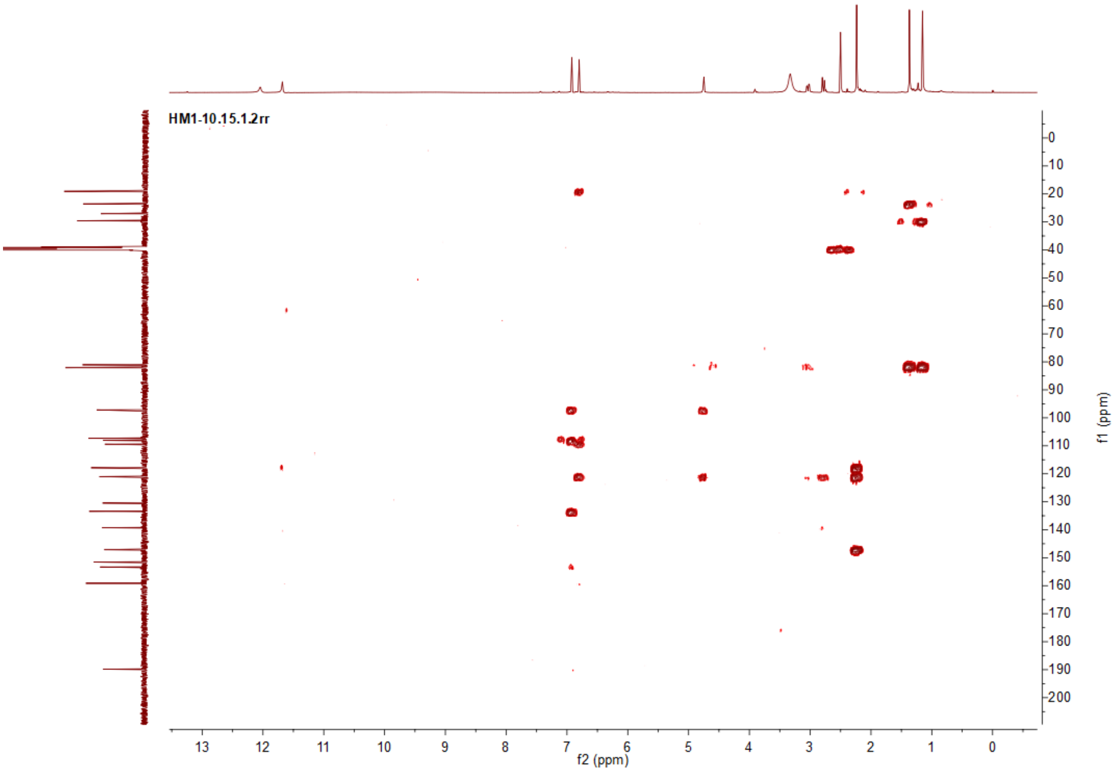

Figure S32. HMBC spectrum of **6**

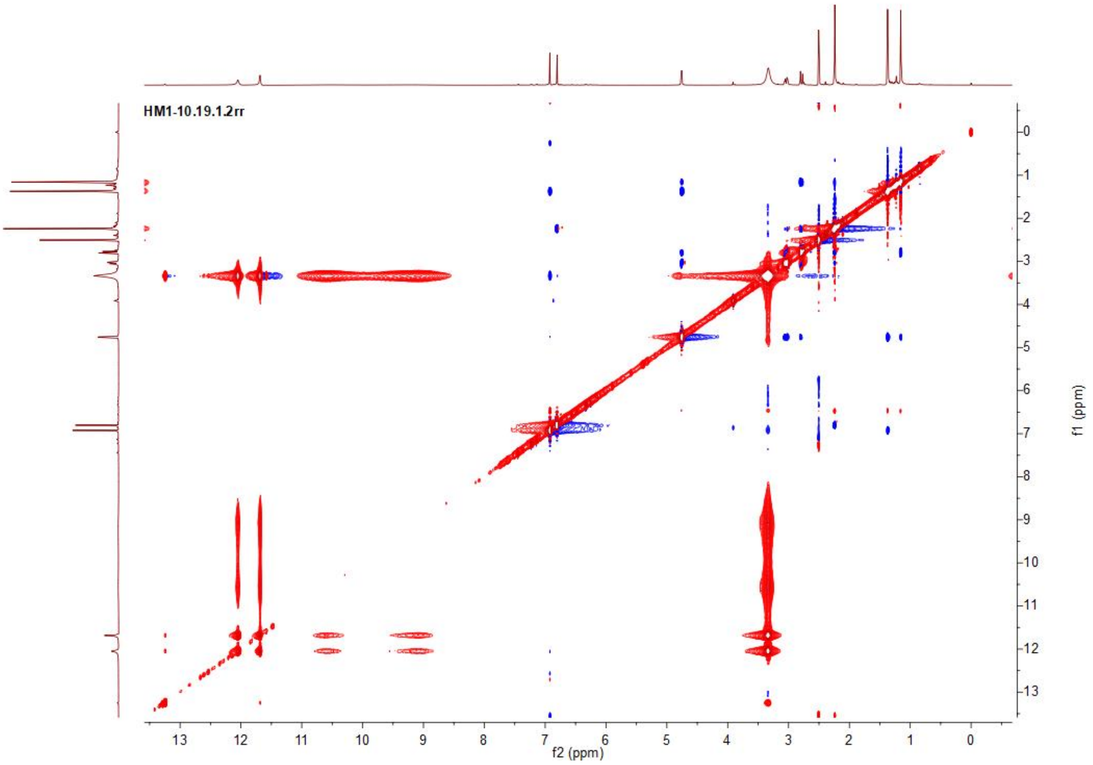

Figure S33. NOESY spectrum of **6**

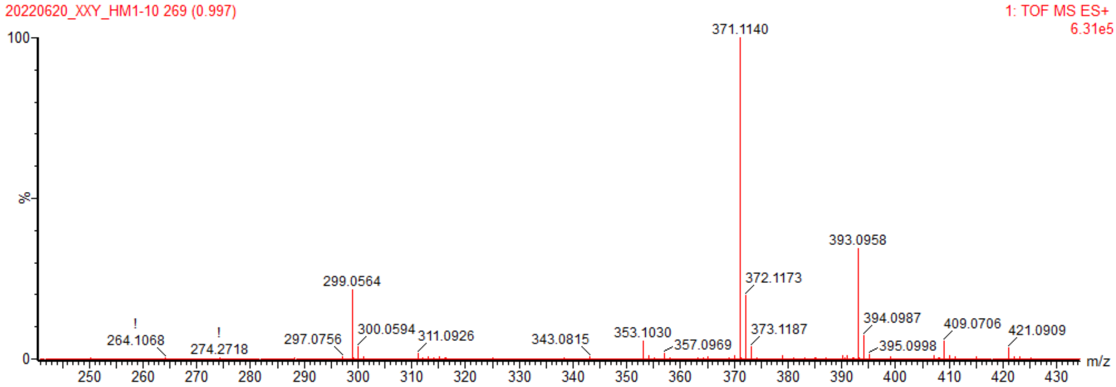

Figure S34. HRESIMS spectrum of **6**

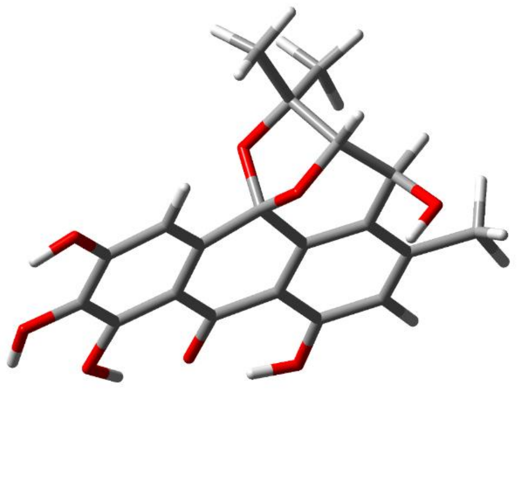

(10*S*,11*S*,12*S*)-**5a**

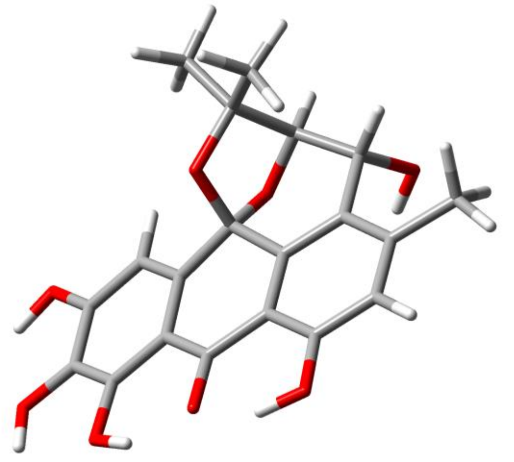

(10*R*,11*R*,12*R*)-**5b**

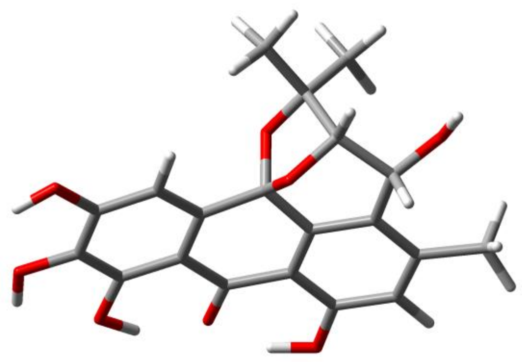

(10*S*,11*R*,12*S*)-**5c**

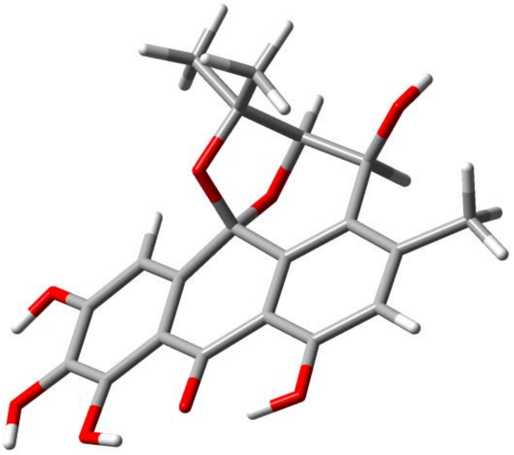

(10*R*,11*S*,12*R*)-**5d**

Figure S35. The optimized structures of conformers **5a-5d** in MeOH at B3LYP/6-31G(d,p) level

Table S1. The Cartesian coordinates of optimized structure of (10*S*,11*S*,12*S*)-**5a**

atom	X	Y	Z
C	3.29258	1.39063	-0.34264
C	2.78199	2.6494	-0.04313
C	1.41716	2.84344	0.21124
C	0.53249	1.73451	0.17354
C	1.0655	0.46064	-0.10793
C	2.41939	0.27448	-0.36234
C	-0.90744	1.91988	0.32146
C	-1.79831	0.79133	0.087
C	-1.29804	-0.50346	-0.18431
C	-3.20116	0.99517	0.06401
C	-4.06013	-0.06751	-0.23033
C	-3.53826	-1.33584	-0.50247

C	-2.15336	-1.55381	-0.46573
O	-1.38097	3.06255	0.60201
C	4.76252	1.21477	-0.6281
O	0.99774	4.09439	0.47249
O	-3.79444	2.18444	0.30787
C	2.91879	-1.11262	-0.73426
C	1.86135	-2.1758	-0.41466
C	1.47619	-2.36467	1.07742
C	0.88236	-3.75671	1.30194
O	0.61541	-1.73075	-0.99118
C	2.57878	-2.05409	2.08742
C	0.17642	-0.77412	-0.03724
O	3.25264	-1.17795	-2.12864
O	-5.41879	0.0832	-0.27255
O	-4.36195	-2.37122	-0.79571
O	0.39684	-1.40002	1.24038
H	3.43256	3.51803	-0.0122
H	-1.76738	-2.54638	-0.66323
H	5.27623	2.17794	-0.68328
H	5.24346	0.6199	0.15926
H	4.90789	0.67681	-1.56977
H	0.01693	4.04244	0.61112
H	-3.06171	2.82919	0.49611
H	3.84937	-1.35023	-0.21282
H	2.13582	-3.11413	-0.89937
H	0.40126	-3.79957	2.28405
H	1.6639	-4.52348	1.26206
H	0.13188	-3.97664	0.53651
H	2.19606	-2.19941	3.10252
H	3.43328	-2.72484	1.9466
H	2.91965	-1.01957	1.99749
H	2.42939	-0.9818	-2.60891
H	-5.63138	1.01188	-0.07259
H	-5.28191	-2.05097	-0.77631

Table S2. The Cartesian coordinates of optimized structure of (10*R*,11*R*,12*R*)-**5b**

atom	X	Y	Z
C	-3.29258	1.39064	-0.34264
C	-2.78199	2.649403	-0.04312
C	-1.41716	2.843442	0.211247
C	-0.53248	1.734509	0.173539
C	-1.0655	0.460644	-0.10792
C	-2.41939	0.274484	-0.36233

C	0.907439	1.91988	0.321459
C	1.798313	0.791326	0.086997
C	1.298044	-0.50346	-0.18431
C	3.201163	0.995171	0.064015
C	4.060134	-0.06751	-0.23033
C	3.53826	-1.33584	-0.50247
C	2.153366	-1.55381	-0.46573
O	1.380971	3.062545	0.602002
C	-4.76251	1.214783	-0.6281
O	-0.99773	4.094385	0.472494
O	3.794439	2.18444	0.307874
C	-2.9188	-1.11262	-0.73425
C	-1.86135	-2.1758	-0.41466
C	-1.47619	-2.36469	1.077416
C	-0.88237	-3.75672	1.301913
O	-0.61541	-1.73074	-0.99118
C	-2.57878	-2.05411	2.087417
C	-0.17642	-0.77413	-0.03724
O	-3.25266	-1.17793	-2.12863
O	5.418789	0.083202	-0.27255
O	4.36195	-2.37122	-0.79571
O	-0.39684	-1.40003	1.240375
H	-3.43255	3.518034	-0.01219
H	1.767377	-2.54639	-0.66323
H	-5.24346	0.619854	0.159206
H	-5.27623	2.177957	-0.68322
H	-4.90789	0.676898	-1.56981
H	-0.01692	4.042447	0.61114
H	3.061721	2.829195	0.49612
H	-3.84937	-1.35023	-0.21281
H	-2.13583	-3.11412	-0.89939
H	-1.66391	-4.52349	1.262052
H	-0.13191	-3.97666	0.536464
H	-0.40125	-3.79959	2.284017
H	-2.91966	-1.01959	1.997503
H	-3.43328	-2.72486	1.946605
H	-2.19605	-2.19944	3.102521
H	-2.42941	-0.98179	-2.6089
H	5.631382	1.01188	-0.07261
H	5.281917	-2.05097	-0.7763

Table S3. The Cartesian coordinates of optimized structure of (10*S*,11*R*,12*S*)-**5c**

atom	X	Y	Z
C	-3.552751	-1.38539	-0.561101
C	-4.093607	-0.145947	-0.211492
C	-3.252756	0.917365	0.120318
C	-1.847685	0.742118	0.103364
C	-1.325796	-0.523817	-0.244086
C	-2.163759	-1.576831	-0.563528
C	-0.976018	1.874895	0.366315
C	0.459269	1.735007	0.154308
C	1.018649	0.487132	-0.185708
C	0.154806	-0.766778	-0.126433
C	1.303532	2.873361	0.178008
C	2.64678	2.737302	-0.18578
C	3.184537	1.50824	-0.557906
C	2.36529	0.350902	-0.515585
C	2.878395	-1.018293	-0.927756
C	1.860161	-2.112223	-0.569548
C	1.517148	-2.331197	0.932993
O	0.400644	-1.41274	1.128899
C	4.619897	1.457148	-1.016796
O	-1.467421	2.990353	0.718954
O	-4.368498	-2.415314	-0.89109
O	-5.454824	-0.019957	-0.217984
O	-3.86203	2.079878	0.437006
O	0.855547	4.097816	0.507762
O	4.150917	-1.287725	-0.342788
C	0.977075	-3.748176	1.13628
C	2.590178	-1.998657	1.964052
O	0.5924	-1.693498	-1.106332
H	-1.760379	-2.54856	-0.819228
H	3.263537	3.630337	-0.197828
H	2.974611	-1.026492	-2.0245
H	2.136271	-3.050599	-1.054877
H	5.250726	0.945387	-0.28572
H	4.715013	0.898628	-1.954335
H	5.00644	2.46759	-1.174737
H	-5.290184	-2.103692	-0.834951
H	-5.6744	0.894756	0.033501
H	-3.133349	2.725975	0.64149
H	-0.113446	3.999873	0.699822
H	4.59644	-1.939446	-0.906195
H	0.507792	-3.828584	2.121679
H	1.78707	-4.483309	1.076784

H	0.228435	-3.98625	0.374575
H	2.928841	-0.966298	1.870517
H	2.166863	-2.140574	2.964384
H	3.455762	-2.65623	1.854681

Table S4. The Cartesian coordinates of optimized structure of (10*R*,11*S*,12*R*)-**5d**

atom	X	Y	Z
C	3.55275	-1.385391	-0.561102
C	4.093606	-0.145948	-0.21149
C	3.252755	0.917364	0.120319
C	1.847685	0.742117	0.103367
C	1.325795	-0.523817	-0.244085
C	2.163758	-1.576831	-0.563529
C	0.976017	1.874894	0.366314
C	-0.459269	1.735008	0.154308
C	-1.01865	0.487132	-0.185708
C	-0.154807	-0.766777	-0.126436
C	-1.303531	2.873362	0.178009
C	-2.646778	2.737304	-0.185781
C	-3.184538	1.508243	-0.557906
C	-2.365292	0.350904	-0.515584
C	-2.878396	-1.018292	-0.927754
C	-1.860162	-2.112223	-0.569546
C	-1.517148	-2.331196	0.932993
O	-0.400649	-1.412737	1.128902
C	-4.619894	1.457151	-1.016804
O	1.467422	2.990355	0.718951
O	4.368497	-2.415314	-0.891093
O	5.454825	-0.019956	-0.217984
O	3.86203	2.079878	0.437007
O	-0.855546	4.097816	0.50777
O	-4.150917	-1.287725	-0.342786
C	-0.977072	-3.748176	1.136276
C	-2.590182	-1.998663	1.964052
O	-0.592399	-1.693496	-1.106331
H	1.760378	-2.548559	-0.819231
H	-3.263535	3.63034	-0.19783
H	-2.974613	-1.026489	-2.024498
H	-2.136271	-3.050598	-1.054875
H	-4.714989	0.898694	-1.954384
H	-5.250716	0.945325	-0.285769
H	-5.00646	2.467594	-1.174683
H	5.290185	-2.103695	-0.834953

H	5.6744	0.894755	0.03351
H	3.133345	2.725976	0.641481
H	0.113447	3.999871	0.69983
H	-4.59641	-1.939498	-0.906158
H	-0.228433	-3.986247	0.374568
H	-0.507784	-3.828585	2.121672
H	-1.787064	-4.483312	1.076781
H	-2.928851	-0.966306	1.870519
H	-3.45576	-2.656243	1.854684
H	-2.166864	-2.140576	2.964384

Functional	Solvent?	Basis Set	Type of Data
mPW1PW91	PCM	6-311G(d,p)	Unscaled Shifts

		DP4+	45.45%	54.55%	0.00%	0.00%	-
Nuclei	sp2?	xperimenta	Isomer 1	Isomer 2	Isomer 3	Isomer 4	Isomer 5
C	x	151.5	137.7	137.5	137.5	137.5	
C	x	133.5	120.9	120.4	120.4	120.4	
C	x	153.7	139	138.7	138.7	138.7	
C	x	107.4	96.4	95.8	95.8	95.8	
C	x	130.2	121.7	122.2	122.2	122.2	
C	x	139.2	129.1	129.4	129.4	129.4	
C	x	123.5	113.9	113.8	113.8	113.8	
C	x	149	141.3	140.6	140.6	140.6	
C	x	118.6	107.8	108.1	108.1	108.1	
C	x	160.4	151	150.3	150.3	150.3	
C	x	108.9	99.7	99.1	99.1	99.1	
C	x	189.7	176.4	176.9	176.9	176.9	
C	x	107.8	99.4	98.7	98.7	98.7	
C		97.2	91.1	91.1	91.1	91.1	
C		61.7	56.3	62.5	62.5	62.5	
C		87.1	79.9	75.8	75.8	75.8	
C		78.4	71.4	75.1	75.1	75.1	
C		29.9	20.5	21.7	21.7	21.7	
C		23.1	13.7	16.4	16.4	16.4	
C		18.5	11.3	15.3	15.3	15.3	
H		6.83	7.12	7.12	7.04	7.04	
H		6.91	7.42	7.42	7.39	7.39	
H		4.52	4.55	4.55	5.61	5.61	
H		4.57	4.62	4.62	4.33	4.33	

Figure S36. DP4+ probabilities (%) for configurations (10*S*,11*S*,12*S*)-**5a** (isomer 1), (10*R*,11*R*,12*R*)-**5b** (isomer 2), (10*S*,11*R*,12*S*)-**5c** (isomer 3), and (10*R*,11*S*,12*R*)-**5d** (isomer 4)

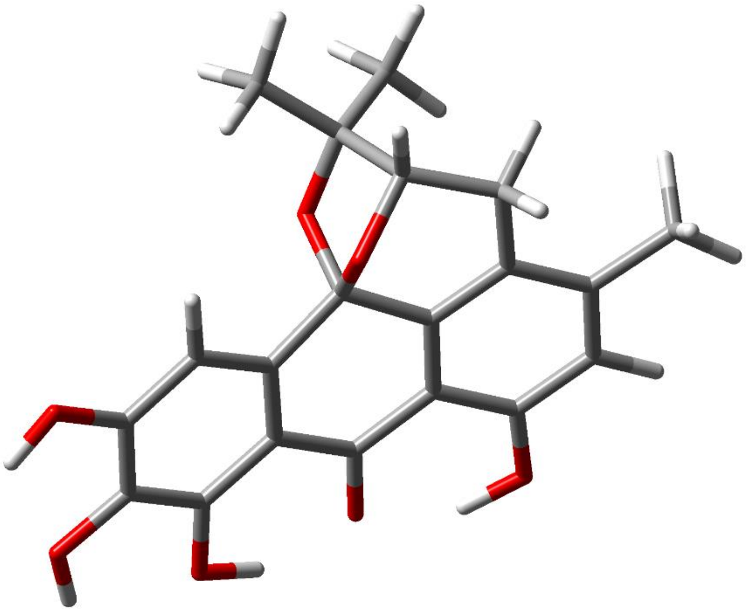

Figure S37. The optimized structure of conformer (10*S*, 12*S*)-**6a** in MeOH at B3LYP/6-31G(d,p) level

Table S5. The Cartesian coordinates of optimized structure of (10*S*, 12*S*)-**6a**

atom	X	Y	Z
C	3.46066	1.20798	-0.53553
C	3.02177	2.48074	-0.17989
C	1.68124	2.72689	0.14059
C	0.74858	1.6579	0.10807
C	1.21155	0.36944	-0.23015
C	2.54321	0.12948	-0.54997
C	-0.67358	1.90094	0.3239
C	-1.62477	0.82159	0.0925
C	-1.1965	-0.48393	-0.24232
C	-3.01653	1.08934	0.13635
C	-3.93567	0.07778	-0.15518
C	-3.48513	-1.20238	-0.49143
C	-2.11178	-1.48351	-0.52163
O	-1.08424	3.0535	0.66025
C	4.9064	0.97751	-0.89469
O	1.32925	3.98828	0.4579
O	-3.54303	2.29547	0.44437
C	2.97151	-1.27065	-0.94703
C	1.86563	-2.29255	-0.67093
C	1.51581	-2.53728	0.82534

C	0.84092	-3.89909	1.00305
O	0.62088	-1.76299	-1.17271
C	2.66302	-2.34687	1.81388
C	0.26958	-0.82776	-0.16775
O	-5.2868	0.29181	-0.13384
O	-4.36816	-2.18882	-0.7831
O	0.50827	-1.51328	1.07566
H	3.71329	3.31742	-0.15385
H	-1.78093	-2.48481	-0.76875
H	5.38506	0.29263	-0.18323
H	4.99847	0.51716	-1.88593
H	5.47183	1.91295	-0.89715
H	0.35447	3.97328	0.63773
H	-2.7723	2.8987	0.62041
H	3.20197	-1.29269	-2.02099
H	3.89234	-1.56748	-0.43429
H	2.05845	-3.22941	-1.19714
H	1.57077	-4.71071	0.90646
H	0.05913	-4.03596	0.24964
H	0.3824	-3.95957	1.9951
H	3.47355	-3.05487	1.60927
H	3.05944	-1.32942	1.76732
H	2.3035	-2.52657	2.83212
H	-5.44608	1.22182	0.10559
H	-5.27033	-1.8276	-0.71381

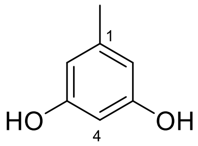

orcinol (**7**)

Orcinol (**7**): Colorless solid; ^{1}H NMR (500 MHz, DMSO-d_6) δ_H: 9.07 (s, 2H, 3, 5-OH), 6.03 (d, J = 2.2 Hz, 2H, H-2, 6), 6.01 (d, J = 2.2 Hz, 1H, H-4), 2.11 (s, 3H, H-7); ^{13}C NMR (125 MHz, DMSO-d_6) δ_C: 158.67 (C-3, 5), 139.62 (C-1), 107.52 (C-2, 6), 100.19 (C-4), 21.67 (C-7).

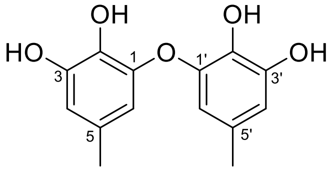

cordyol C (**8**)

Cordyol C (**8**): Yellowish solid; ^{1}H NMR (500 MHz, DMSO-d_6) δ_H: 6.45 (d, J = 2.1 Hz, 1H, H-4), 6.20 (dd, J = 1.9, 0.9 Hz, 1H, H-6), 6.23 (m, J = 1.9 Hz, 1H, H-4'), 6.14 (m, 1H, H-6'),

6.04 (t, J = 2.2 Hz, 1H, H-2'), 2.15 (s, 3H, H-7), 2.11 (s, 3H, H-7'); ^{13}C NMR (125 MHz, DMSO-d_6) δ_C: 159.18 (C-1'), 158.26 (C-3'), 146.86 (C-3), 143.21 (C-1), 139.49 (C-5'), 135.3 (C-2), 127.71 (C-5), 112.73 (C-6), 112.66 (C-4), 109.77 (C-4), 108.00 (C-6'), 100.76 (C-2'), 21.24 (C-7'), 20.48 (C-7).

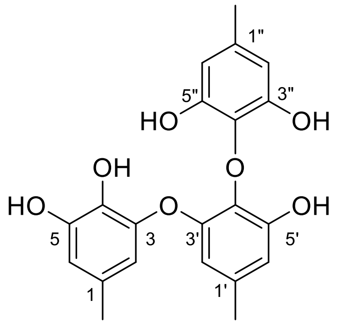

Aspergilol C (**9**)

Aspergilol C (**9**)**:** Brown solid; ^{1}H NMR (500 MHz, DMSO-d_6) δ_H: 6.35 (br s, 1H, H-6'), 6.21 (br s, 4H, H-2, 6, 2", 6"), 5.78 (br s, 1H, H-2'), 2.14 (s, 3H, H-7"), 2.07 (s, 3H, H-7'), 1.99 (s, 3H, H-7).

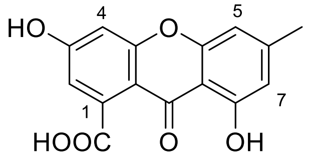

calyxanthone (**10**)

Calyxanthone (**10**)**:** Yellowish solid**;** ^{1}H NMR (500 MHz, DMSO-d_6) δ_H: 12.51 (s, 1H, 8-OH), 6.87 (s, 1H, H-2), 6.81 (s, 1H, H-4), 6.75 (s, 1H, H-5), 6.61 (s, 1H, H-7); ^{13}C NMR (125 MHz, DMSO-d_6) δ_C: 179.3 (C-9), 169.7 (C-10), 164.6 (C-3), 160.7 (C-8), 157.7 (C-4a), 155.2 (C-5a), 148.4 (C-6), 112.3 (C-4), 111.1 (C-7), 108.4 (C-8a), 107.2 (C-5), 105.8 (C-1a), 102.4(C-2), 22.0 (6-CH_3).

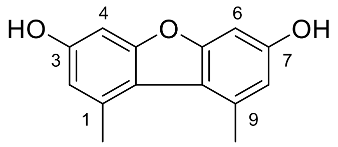

3,7-dihydroxy-1,9-dimethyldibenzofuran **(11)**

3,7-Dihydroxy-1,9-dimethyldibenzofuran **(11):** Light red solid; ^{1}H NMR (500 MHz, DMSO-d_6) δ_H: 6.74 (d, J = 2.2 Hz, 2H, H-4, 6), 6.56 (d, J = 2.2 Hz, 2H, H-2, 8), 2.74 (s, 6H, H-10, 11); ^{13}C NMR (125 MHz, DMSO-d_6) δ_C: 157.0 (C-4a, 5a), 155.7 (C-3, 7), 131.4 (C-1, 9), 115.5 (C-9a, 9b), 114.0 (C-2, 8), 95.6 (C-4, 6), 24.4 (C-10, 11).

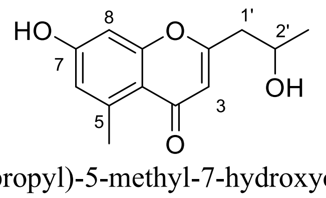

2-(2'-hydroxypropyl)-5-methyl-7-hydroxychromone (**12**)

2-(2'-Hydroxypropyl)-5-methyl-7-hydroxychromone (**12**): Yellowish solid**;** ^{1}H NMR (500 MHz, CD_3OD) δ_H: 6.66 (d, J = 2.3 Hz, 1H, H-8); 6.64 (d, J = 2.3 Hz, 1H, H-6), 6.06 (s, 1H, H-

3), 2.71 (s, 3H, CH_3-11), 1.27 (d, J = 6.2 Hz, 3H, CH_3-3'); ^{13}C NMR (125 MHz, CD_3OD) δ_C: 182.5 (C-4), 167.6 (C-2), 163.6 (C-7), 162.0 (C-9), 144.2 (C-5), 118.5 (C-3), 116.3 (C-10), 113.0 (C-6), 102.2 (C-2), 66.9 (C-2'), 44.7 (C-1'), 24.0 (C-3'), 23.7 (C-11).

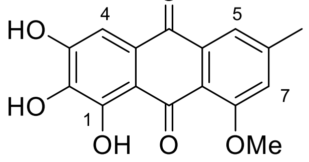

evariquinone (**13**)

Evariquinone (**13**): Orange solid; ^{1}H NMR (500 MHz, DMSO-d_6) δ_H: 7.21 (s, 1H, H-4), 7.08 (d, J = 1.0 Hz, 1H, H-5), 6.89 (d, J = 1.0 Hz, 1H, H-7), 3.17 (s, 3H, 8-OCH_3), 2.29 (s, 3H, H-11); ^{13}C NMR (125 MHz, DMSO-d_6) δ_C: 190.4 (C-9), 180.0 (C-10), 161.3 (C-8), 152.1 (C-3), 151.4 (C-1), 148.1 (C-6), 138.9 (C-2), 132.8 (C-10a), 124.6 (C-4a), 123.3 (C-5), 120.0 (C-7), 113.2 (C-8a), 109.7 (C-9a), 109.2 (C-4), 48.6 (8-OCH_3), 21.6 (C-11).
